# Influences of Seasonal Monsoons on the Taxonomic Composition and Diversity of Bacterial Community in the Eastern Tropical Indian Ocean

**DOI:** 10.3389/fmicb.2020.615221

**Published:** 2021-01-26

**Authors:** Ping Gao, Guangxun Du, Duo Zhao, Qinsheng Wei, Xuelei Zhang, Lingyun Qu, Xianzhe Gong

**Affiliations:** ^1^MNR Key Laboratory of Marine Eco-Environmental Science and Technology, First Institute of Oceanography, Ministry of Natural Resources (MNR), Qingdao, China; ^2^Laboratory for Marine Ecology and Environmental Science, Pilot National Laboratory for Marine Science and Technology (Qingdao), Qingdao, China; ^3^Institute of Marine Science and Technology, Shandong University, Qingdao, China

**Keywords:** seasonal monsoons, structure of bacterioplanktonic community, Eastern Tropical Indian Ocean, 16S rRNA, co-occurrence patterns

## Abstract

The Indian Ocean is characterized by its complex physical systems and strong seasonal monsoons. To better understand effects of seasonal monsoon-driven circulation on the bacterioplanktonic community structure in surface waters and the bacterial distribution response to vertical stratification, patterns of seasonal, and vertical distribution of bacterial communities in the Eastern Tropical Indian Ocean were investigated using 16S rRNA gene profiling. Water samples were collected during the Southwest monsoon (from June to August), the fall inter-monsoon (from October and November) and the Northeast monsoon (from December to January), respectively, onboard during three cruises from July 2016 to January 2018. Surface bacterioplankton communities in these three seasons and in the upper water (3–300 m with six depths) during the Northeast monsoon contained a diverse group of taxa, mainly Proteobacteria, Cyanobacteria, Actinobacteria, Bacteroidetes, and Chloroflexi. Redundancy discriminant analysis (RDA) uncovered that temperature, salinity, and dissolved oxygen (DO) were crucial environmental parameters that affected the structure of bacterial community in overall surface samples. However, significant differences in the composition of the bacterial community are likely due to changes in concentrations of salinity during the fall inter-monsoon, while phosphate for both the Southwest monsoon and the Northeast monsoon. Pearson's analysis revealed that the seasonal variation rather than the vertical variation of environmental factors had a more significant impact on the composition of bacterial community. In addition, a clear seasonal pattern of bacterial co-occurrence showed that inter-taxa associations during the fall inter-monsoon were closer than during the Northeast monsoon and the Southwest monsoon. Overall, our results implied clear differences in the composition of bacterial community, with more pronounced seasonal variation compared to the vertical variation in response to environmental changes.

## Introduction

Patterns of diversity and the composition of bacteria in seawater are critical for exploring the evolutionary and ecological processes that form current biodiversity, and monitor the response of marine ecosystems to environmental changes (Suh et al., 2014). The Indian Ocean, which lies to the south of the largest continent, Eurasia, is characterized by its complex physical dynamics and variable systems (Qian et al., 2018). Huge thermal differences between the land and the ocean lead to the prevailing monsoon in the Indian Ocean. The monsoon is characterized as two distinct seasons, separated by two transition (inter-monsoon) periods: the Southwest monsoon from June to September, and the Northeast monsoon from December to March. The spring transition (pre-monsoon) occurs in April and May, and the fall transition (post-monsoon) occurs in October and November (Fazeli et al., 2013). Impacts of the monsoon in the northern Indian Ocean are apparently supported by the severe response of boundary current in the basin (Hood et al., 2017). Monsoons and monsoon-driven circulation have the greatest effect on the surface water (Schott and McCreary, 2001). During the Southwest monsoon, the Southwest Monsoon Current south of Sri Lanka and the South Equatorial Current are the two important currents that influence the Eastern Tropical Indian Ocean. The South Equatorial Current is located in the south of 10°S from May to June, and its velocity is weak. From July to September, it gradually extends northward to the area of 5°S, and the velocity increases with a maximum of 0.7 m·s^−1^. The change of the South Equatorial Current may relate to the northward movement of Southeast trade wind regime (Schott and McCreary, 2009). During the Northeast monsoon, the Northeast Monsoon Current and South Equatorial Countercurrent significantly affect the Eastern Tropical Indian Ocean. The Northeast Monsoon Current is enhanced in December and peaked in January with a maximum velocity of 0.5 m·s^−1^. In addition, a particular phenomenon, singular to the Indian Ocean owing to the semiannual eastward winds along the equator, is the occurrence of strong eastward surface jets (Wyrtki, 1973) during the transition seasons between the monsoons, i.e., from April to June and from October to December.

The monsoon circulation is predominately wind-driven, although in some locations it is modified by heat and fresh-water fluxes (Schott and McCreary, 2001). As previously reported, heavy precipitation during the Northeast monsoon is correlated with the change of hydrological conditions and associated microbial communities (Chénard et al., 2019). Seasonal changes on bacterial communities have been reported in several studies (Lu et al., 2015; Bandekar et al., 2018), although logistical problems of periodically collecting samples make “time-series” studies less common. Furthermore, surface bacteriological studies, such as the diversity, the composition of community, and the taxonomy in the Eastern Tropical Indian Ocean, influenced by seasonal monsoons are rare. Therefore, more information on this topic would be a valuable piece of the puzzle.

The vertical stratification of microbial communities in the ocean was first identified in the case of phytoplankton (Olson et al., 1990; Moore et al., 1998). Later on, studies using molecular probes as genetic markers for uncultivated microbial groups uncovered many more examples (Treusch et al., 2009; AgoguÉ et al., 2011; Bandekar et al., 2018; Qian et al., 2018). In three distinct environments of the Pacific Ocean, the microbial diversity and richness increased along the depth through the water column (Walsh et al., 2015). Additionally, in the Arabian Sea's oxygen minimum zone (OMZ), a clear vertical partitioning of bacterial communities, between the surface (surface and deep chlorophyll maxima) and deeper waters was observed. In the tropical East Indian Ocean, a strong vertical temperature gradient occurs between 50 and 150 m, indicating the presence of a strong thermocline below the mixed layer (Xuan et al., 2012). However, our knowledge about vertical distribution patterns of the bacterial community in response to the stratification in the Eastern Tropical Indian Ocean remains limited.

Previous reports have indicated that microbial community structures in diverse marine environments differ from one to another (Delong et al., 2006; Zinger et al., 2011). For example, adjacent marine water masses with dissimilar environmental parameters have diverse microbial groups (AgoguÉ et al., 2011). Similarly, there are distinct patterns of bacterial compositions in South and North Pacific Oceans (Suh et al., 2014). Therefore, uncovering the influence of biogeochemical characteristics on the community is crucial to understand the driver of the microbial distribution (Bianchi et al., 2018). Salinity, nitrogen and sulfate all have significant impacts on halophilic taxa (Bouvier and del Giorgio, 2002), denitrifiers (Zheng et al., 2014), and sulfate-reducing prokaryotes (Guo et al., 2018), respectively. Additionally, dissolved oxygen (DO) is a critical environmental factor that determines the distribution of anammox bacteria in the water column (between 0 and 2,000 m, with seven depths) (Qian et al., 2018). Besides the effect of change of geochemical conditions, the microbial communities could also be affected by other physiochemical conditions. In the Eastern Tropical Indian Ocean, a reversal of wind directions causes the seasonal variation in the surface circulation, and the current could directly change the microbial communities. In addition, the seasonal change of hydrological condition, especially in surface water due to the heavy precipitation caused by monsoon may have profound impacts on the microbial communities. However, the response of the composition and function of bacterial communities to these changing environmental conditions remain unclear, and the vertical structure of microbial assemblages associated with strong stratification in the upper water also needs to be explored.

In this study, we aim to (1) assess the composition, as well as the co-occurrence pattern of surface bacterial communities influenced by seasonal monsoons, and (2) reveal how the bacterial diversity and predicted functional genes based on 16S rRNA amplicon sequences related to nitrogen (N), sulfur (S), and oxygen (O) cycling are affected by various environmental factors.

## Materials and Methods

### Seawater Sampling and Physicochemical Characteristics

**Figure 1:**
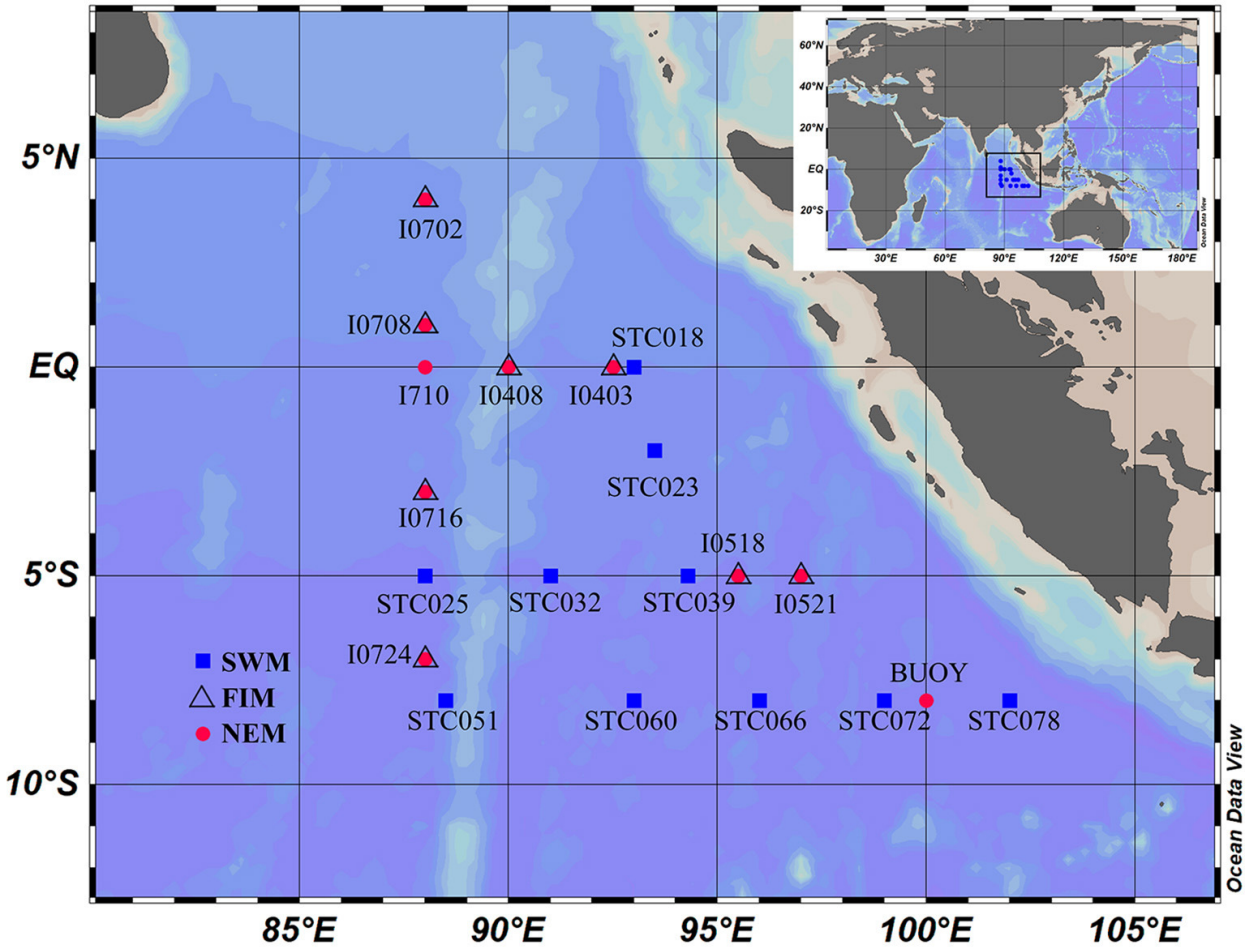
Sampling sites in the Eastern Tropical Indian Ocean. Sites marked by both triangle and red circle indicate that both the fall inter-monsoon and the Northeast monsoon samples are available. SWM, Southwest monsoon; FIM, fall inter-monsoon; NEM, Northeast monsoon.

Surface water were collected from the Eastern Tropical Indian Ocean, an area heavily affected by seasonal monsoons. Water samples were taken in 10 sites using during the cruise by the R/V “*Hai Ce 3301*” from July to August 2016 (Southwest monsoon), 8 sites during two cruises by the R/V “*Xiang Yang Hong 01*” from October to November 2016 (fall inter-monsoon), and the R/V “*Xiang Yang Hong 18*” from December to January 2017–2018 (Northeast monsoon), respectively (Figure 1). In addition, upper waters of two sites (I710 and BUOY) with six depths (3, 30, 75, 100, 150, and 300 m) were sampled during the Northeast monsoon. Water samples were collected by using Niskin bottles (General Oceanic Inc.), which were mounted to a Sea-Bird SBE-911 Plus V2 Conductivity-Temperature-Depth (CTD) system (Sea-Bird Scientific, USA).

Five liters of seawater for each sample were immediately filtered through 0.22 μm pore-size polycarbonate filter (Waterman, France) with vacuum. Filters with biomass were then transferred to cryovials and stored at −80°C until DNA extraction. The temperature, salinity, and depth were recorded *in situ* from sensors fitted onto CTD recorders. pH was recorded using a pH meter (ORION STAR A211), and DO was measured on board following the Winkler's titration procedure (Grasshoff et al., 1999), and total organic carbon (TOC) was analyzed according to the report by Wang et al. (2012). Methods and procedures for quantifying nutrients including nitrate, nitrite, ammonia, and phosphate were based on those described by Parsons et al. (1984). Dissolved inorganic nitrogen (DIN) was calculated as the total of NO_3_-N, NO_2_-N, and NH_4_-N. All the environmental factors were measured in triplicates.

### DNA Extraction, Amplification, and Sequencing

Total DNA was extracted by the water DNA kit (OMEGA BioTek Inc., Doraville, GA, USA) following the manufacturer's procedures. The DNA quality was checked by 1% (w/v) agarose gel electrophoresis. Primers 341F and 806R (Michelsen et al., 2013) targeting the bacterial 16S rRNA gene were used to amplify the V3-V4 hyper-variable regions. The PCR products were sequenced on an Ion S5™ XL platform by Novogene Bioinformatics Technology Co., Ltd (Beijing, China).

### Amplicon Sequence Analysis

Sequences were separated according to their unique barcodes, and trimmed by removing the barcodes and primer sequences, then raw sequences generated. Afterwards, raw single-end reads were quality filtered (Martin, 2011), denoised, merged, and chimera checked using the UCHIME algorithm (Edgar et al., 2011). Chimera sequences were removed and operational taxonomic units (OTUs) were clustered at 97% identity using UPARSE software (V7.0.1001) (Edgar, 2013). A representative sequence for each OTU was selected for further annotation using the Silva Database (Quast et al., 2012) based on the Mothur algorithm (Schloss et al., 2009). For samples from surface water during different seasons, 2,212,401 reads were retrieved from 26 samples, and 2,096,468 sequences were left after quality control, and rarefied at the depth of 48,284 for downstream analysis. In addition, 1,015,068 reads were retrieved from 12 samples from upper waters of two sites with different depths during the Northeast monsoon, and 963,416 sequences were left after quality control, and rarefied at the depth of 59,201. Functional assignments were predicted using Tax4fun2 (https://github.com/bwemheu/Tax4Fun2) based on the OTUs with Ref99NR database.

### Bioinformatics and Statistical Analysis

Sampling sites were plotted by the Ocean Data View (ODV) software (https://odv.awi.de), and the concentrations of environmental factors at each site were analyzed by the data-interpolating variational analysis gridding method to draw contour distribution maps. In order to estimate the bacterial alpha diversity, Chao (1984) indices based on the OTUs were calculated using QIIME (Version1.7.0) and displayed using “vegan” package in R (Version 2.15.3) with the Wilcox test. Statistical differences of community structure among groups (from different locations and water layers) were conducted by the analysis of similarity (ANOSIM) test. Unweighted pair-group method with arithmetic mean (UPGMA) (Li and Xu, 2007) clustering was carried out to reveal the distance matrix using average linkage and calculated by QIIME software (Version 1.7.0). Bacterial taxa with significantly different relative abundance among samples from different seasons were identified by a linear discriminant analysis effect size (LEfSe). A redundancy discriminant analysis (RDA) was carried out to identify the correlation between bacterial groups and environmental parameters. To simplify the complex relationship between bacterial taxa/predicted functional genes and physicochemical parameters, the Pearson's correlation coefficient was calculated and displayed by “ggcor” package (https://github.com/houyunhuang/ggcor) in R. The bacterial groups or predicted functional genes were clustered with UPGMA in R “pheatmap” package (https://github.com/raivokolde/pheatmap). The predicted functional genes belong to the same enzyme were clustered as a whole by the mean value of Pearson's correlation coefficient. Spearman's analysis was performed to explore the relationship between bacterial diversity and biogeochemical factors. Network analysis based on Spearman's correlation was calculated by molecular ecological network analyses pipeline (MENAP) for its accuracy to provide remarkable solutions to issues of noise associated with high-throughput sequencing data with a cutoff of 0.97 determined by random matrix theory (Deng et al., 2012).

### Sequence Data Accession Numbers

Raw sequences were deposited into the National Center for Biotechnology Information (NCBI) Sequence Read Archive under the BioProject accession PRJNA657710 and the SRA accession SRR12474819.

## Results

### Seasonal and Vertical Variations of Environmental Factors

**Figure 2:**
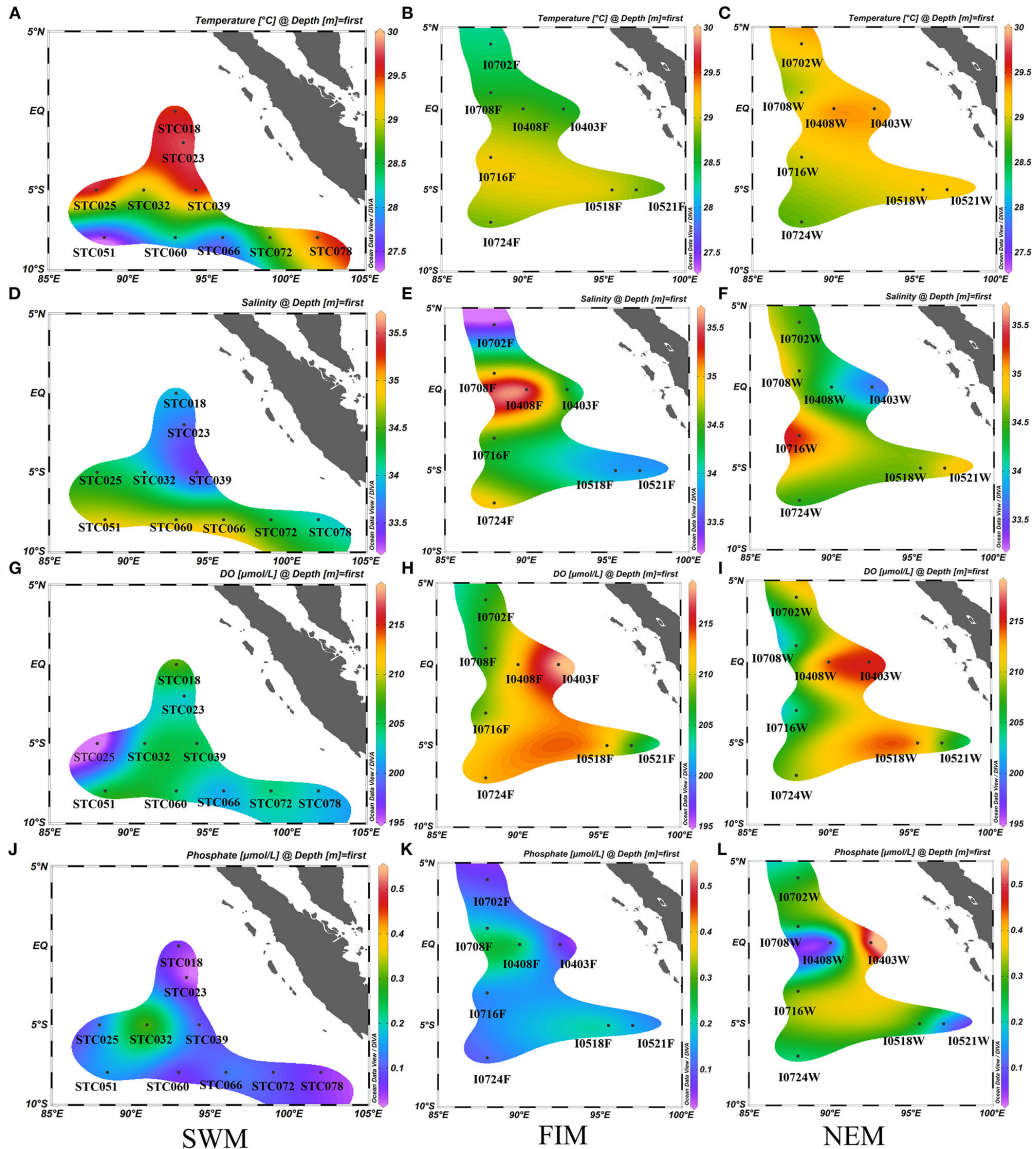
Seasonal and horizontal variations of temperature (°C) **(A–C)**, salinity **(D–F)**, dissolved oxygen (DO, μmol/L) **(G–I)** and phosphate (μmol/L) **(J–L)** in surface water in the Eastern Tropical Indian Ocean) during the Southwest monsoon (SWM) **(A,D,G,J)**, the fall inter-monsoon (FIM) **(B,E,H,K)** and the Northeast monsoon (NEM) **(C,F,I,L)**, respectively.

Detailed descriptions of environmental parameters including temperature, salinity, pH, DO, DIN, phosphate, and TOC in surface water in three seasons (the Southwest monsoon, the fall inter-monsoon and the Northeast monsoon), and in upper water (between 3 and 300 m, with six depths) during the Northeast monsoon are presented in Supplementary Table 1. The surface water temperature at the equator and five degrees south during the Southwest monsoon (Figure 2A) was higher than other seasons (Figures 2B,C). The surface water salinity south of the equator was higher than that near the equator areas during the Southwest monsoon (Figure 2D), while the salinity near the equator increased during the fall inter-monsoon (Figure 2E). Generally, the salinity south of the equator was higher than that north of the equator during the Northeast monsoon (Figure 2F). In terms of DO, it was lower during the Southwest monsoon (Figure 2G) than during the Northeast monsoon (Figure 2I) and the fall inter-monsoon (Figure 2H), and was highest at the equator. The average concentration of phosphate in surface water was highest during the Northeast monsoon (Figure 2L), followed by the fall inter-monsoon (Figure 2K) and the Southwest monsoon (Figure 2J).

In addition, the vertical distribution pattern of the above factors at the station I710 and BUOY during the Northeast monsoon is shown in Supplementary Table 1. It is noteworthy that the obvious thermocline stratification along 75–150 m caused a significant change in environmental parameters, especially for temperature, DO and nutrients. As expected, temperature and DO gradually decreased with depth and dropped sharply from 100 to 300 m along a vertical gradient. However, nutrients (phosphate and nitrogen) concentrations suddenly increased below the thermocline. TOC at the station BUOY, at each measured depth was much lower than the corresponding layer in I710, which might be attributed to the river input at the boundary of the Eastern Indian Ocean.

### Bacterial Diversity and Richness

**Figure 3:**
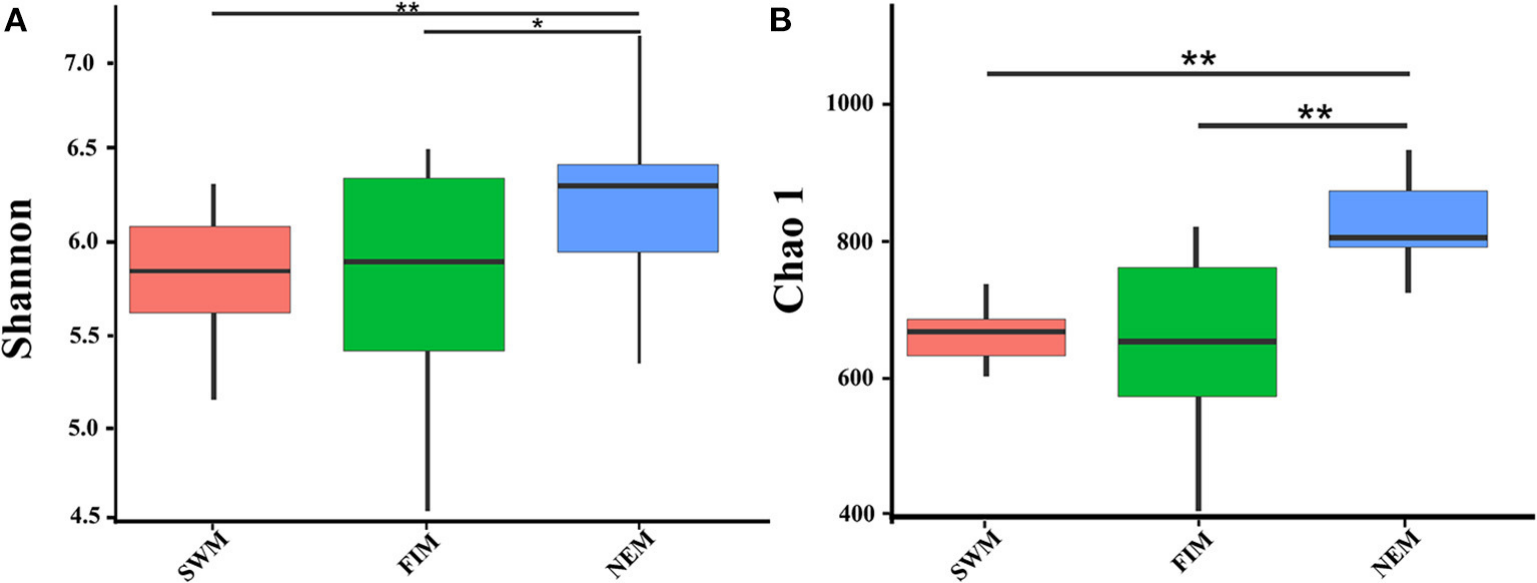
The bacterial diversity of surface water in the Eastern Tropical Indian Ocean during the Southwest monsoon (SWM), the fall inter-monsoon (FIM) and the Northeast monsoon (NEM), respectively. Indices of alpha diversity were shown as **(A)** Shannon, and **(B)** Chao 1. There were significant differences between seasons in each sampling area by Wilcox test (**p < 0.01; *p < 0.05).

There were 3,150 and 2,773 OTUs at the 0.03 distance level in samples from surface water during different seasons (26 samples) and from upper waters with different depth during the Northeast monsoon (12 samples), respectively. The diversity and richness of the bacterial communities in surface or upper water of the Eastern Tropical Indian Ocean were characterized by the Shannon and Chao1 indices, respectively (Figure 3, Supplementary Table 2). Bacterial diversity (Shannon index) in surface water differed during the fall inter-monsoon, the Northeast monsoon, and the Southwest monsoon, ranging from 4.70 to 6.41, from 5.55 to 6.73, and from 5.44 to 6.17, respectively, while the richness ranged from 506.54 to 894.87, from 822.61 to 1099.40, and from 693.30 to 807.66, respectively. The highest and the lowest diversity and richness in surface water were recorded during the Northeast monsoon (I0716W) and the fall inter-monsoon (I0521F), respectively (Supplementary Table 2). There were significant differences in both the Shannon and Chao1 indices among different seasons (Wilcox test). On average, the Northeast monsoon samples had a higher Shannon index than both the fall inter-monsoon samples (*p* = 0.012) and the Southwest monsoon samples (*p* = 0.0013). Samples from the Northeast monsoon were also significantly higher in terms of average richness, than from the fall inter-monsoon (*p* < 0.01) and the Southwest monsoon (*p* < 0.01) (Supplementary Table 3). In addition, pronounced variations in diversity and richness across vertical gradients of the water column in upper water (between 3 and 300 m, with six depths) were also observed during the Northeast monsoon (Supplementary Table 2). The highest diversities at station I710 and BUOY were recorded from 75 m and surface samples, respectively, and the highest richness was recorded from 150 and 300 m, respectively.

### Bacterial Taxonomic Assignment and Community Composition

**Figure 4:**
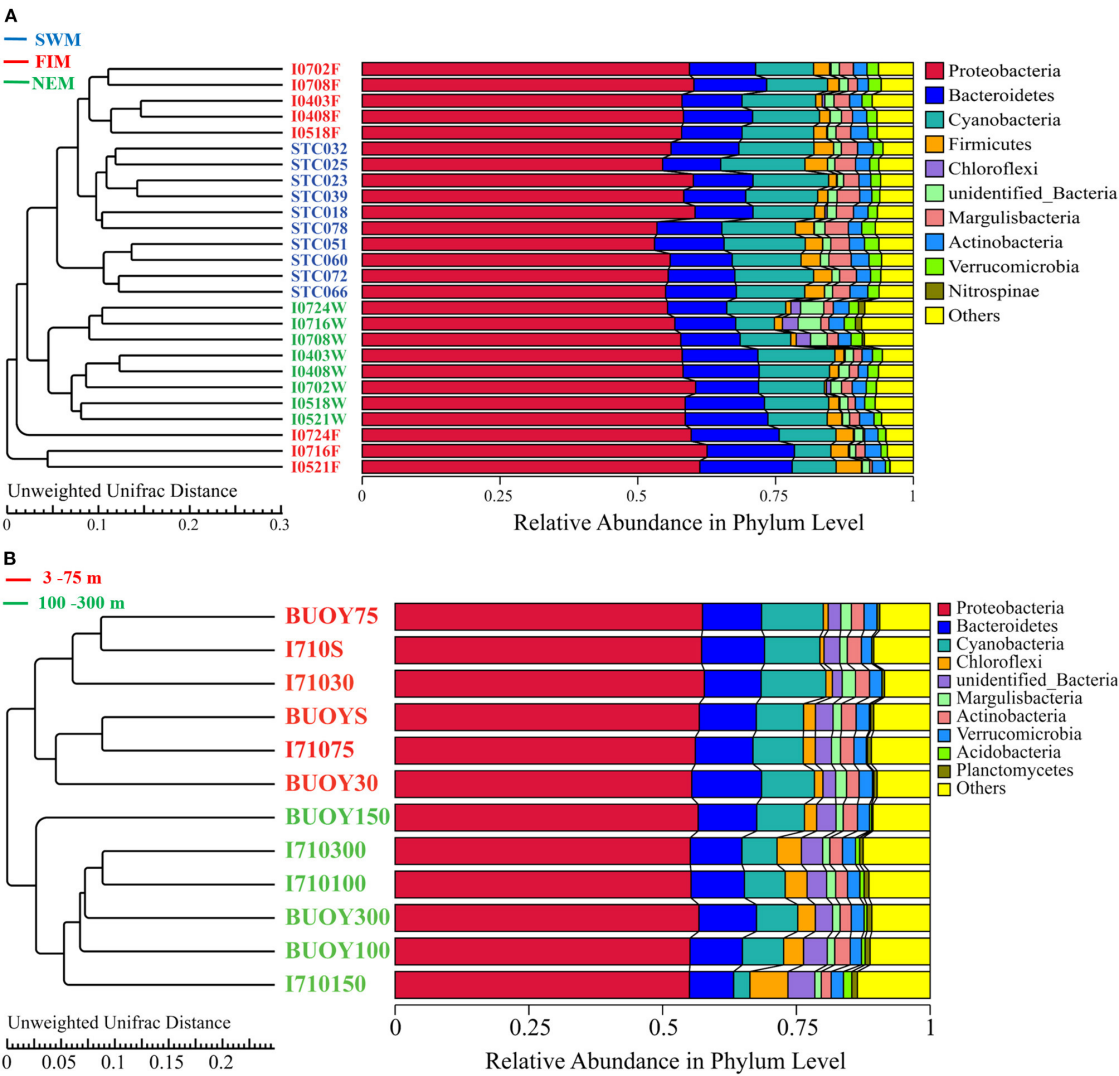
UPGMA analysis based on Unweighted-Unifrace distance of surface samples during **(A)** the Southwest monsoon (SWM), the fall inter-monsoon (FIM) and the Northeast monsoon (NEM), respectively, and **(B)** in upper water during the NEM.

Bacterial sequences were classified into 152 families and 274 genera, 138 families and 265 genera, and 165 families and 320 genera in samples from the Southwest monsoon (*n* = 10), the fall inter-monsoon, (*n* = 8) and the Northeast monsoon (*n* = 8), respectively. There were 182 families and 366 genera in upper water (*n* = 12). All samples were dominated by Proteobacteria, followed by Cyanobacteria and Bacteroidetes (Figure 4). A resolved phylogenetic classification displayed diverse community structure over horizontal, seasonal, and vertical scales for all the samples. Alphaproteobacteria, Gammaproteobacteria, and unidentified Cyanobacteria were the three most abundant classes in all samples. In terms of seasonality, the abundance of Gammaproteobacteria was highest during the fall inter-monsoon and lowest during the Northeast monsoon, whereas the unidentified Cyanobacteria displayed the inverse trend. For Alphaproteobacteria, the relative abundance remained constant between seasons. Interestingly, the- class Nitriliruptoria with low relative abundance, belonging to Actinobacteria, displayed obvious seasonality and was hardly detected during the Southwest monsoon, but widely distributed during the fall inter-monsoon and the Northeast monsoon (Supplementary Figure 1). Spatially, Proteobacteria in I0521F, I0716F, and I0724F (south of the equator) were much more abundant than in stations located north of the equator during the fall inter-monsoon. While, the trend was reversed in Cyanobacteria (Figure 4A). In upper water during the Northeast monsoon, the relative abundance of Proteobacteria generally increased with depth, and the highest abundance of photosynthetic Cyanobacteria and Chloroflexi are mainly located between 30 and 75 m, indicating apparent dissimilarities in bacterial community composition along the vertical water column (Figure 4B).

### Similarity and Dissimilarity Analysis Among Bacterial Communities

UPGMA analysis revealed that samples from the Southwest monsoon and the Northeast monsoon formed two clusters, while samples from the fall inter-monsoon were divided into two different groups (Figure 4A): one grouped by samples from north of the equator, except I0518F, and the other from south of the equator. ANOSIM results also demonstrated that there are greater significant differences between the bacterial community composition of samples from north or south of the equator, than within these groups (*R* = 0.788, *p* = 0.001) (Supplementary Table 4). Clear patterns of seasonal as well as horizontal partitioning during the fall inter-monsoon were observed in the sampling area. Along the vertical gradient, samples in upper waters during the Northeast monsoon were divided into two significant clusters: one from 3 to 75 m, and the other from 100 to 300 m (Figure 4B). ANOSIM results also confirmed that the bacterial taxa between the two clusters varied significantly from each other during the Northeast monsoon (*R* = 0.208, *p* = 0.002) (Supplementary Table 4). The vertical pattern of samples showed clear dissimilarities of bacterial assembly in terms of depths.

**Figure 5:**
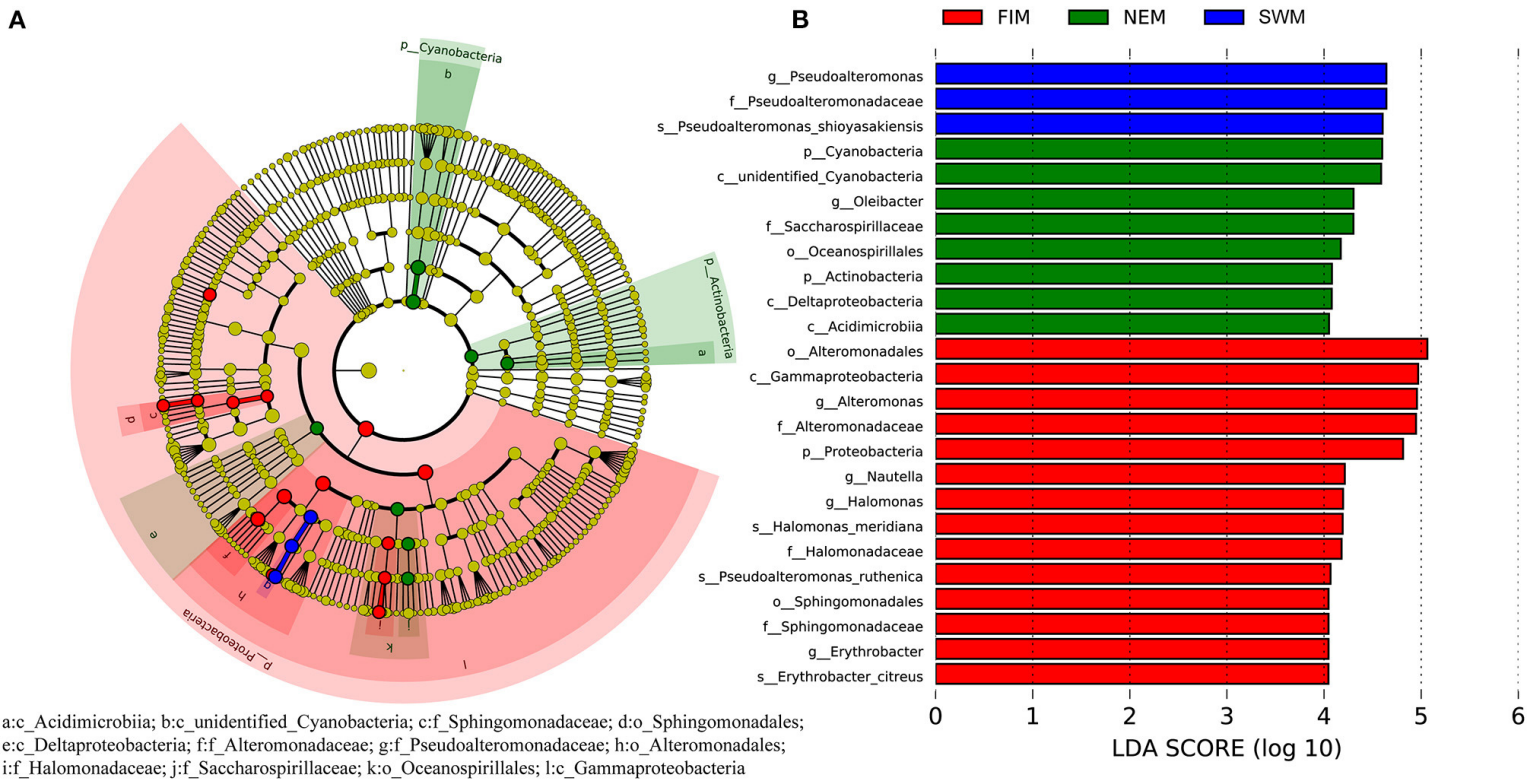
Cladogram based on LEfSe analysis showing the differentially abundant bacteria among samples during the Southwest monsoon (SWM), the fall inter-monsoon (FIM), and the Northeast monsoon (NEM), respectively **(A)**, with a linear discriminant analysis (LDA) threshold of 4 **(B)**. The bacteria with significantly different abundance at different taxonomic levels among samples were highlighted by colored circles and shadings.

To discover bacterial assemblage with significant differences among seasons, LEfSe was used to identify the differentially abundant taxa of each sample (Figure 5A). Samples with a linear discriminant analysis (LDA) score higher than four were retained (Figure 5B). Results demonstrated that Pseudoalteromonadaceae (order Alteromonadales, class Gammaproteobacteria) was more abundant during the Southwest monsoon, families of Alteromonadaceae and Halomonadaceae, both affiliated to Gammaproteobacteria, were more enriched during the fall inter-monsoon, whereas families Acidimicrobiia belonging to Actinobacteria and unidentified Cyanobacteria preferred during the Northeast monsoon (Figure 5).

LEfSe analysis was also performed to identify the special taxa with significantly different abundance among samples at a spatial scale (both horizontal and vertical). Based on the UPGMA analysis, it revealed that samples located north or south of the equator during the fall inter-monsoon displayed distinct dissimilarities in the composition of bacterial community. Here, we found that the order Synechococcales and unidentified Cyanobacteria in class level, both affiliated to phylum Cyanobacteria, unidentified Alphaproteobacteria in order level, and the order Oceanospirillales were dominant in samples from north of the equator. However, the family Alteromonadaceae was much more abundant in samples south of the equator (32.06%) than north of the equator (8.28%) (Supplementary Figure 2A). In the vertical water column, it is interesting to note that families of the unidentified Cyanobacteria and unidentified Alphaproteobacteria preferred depths < 100 m while the class Gammaproteobacteria was significantly enriched at depths ≥100 m (Supplementary Figure 2B).

### Relationship Between Bacterial Taxa and Environmental Characteristics

**Figure 6:**
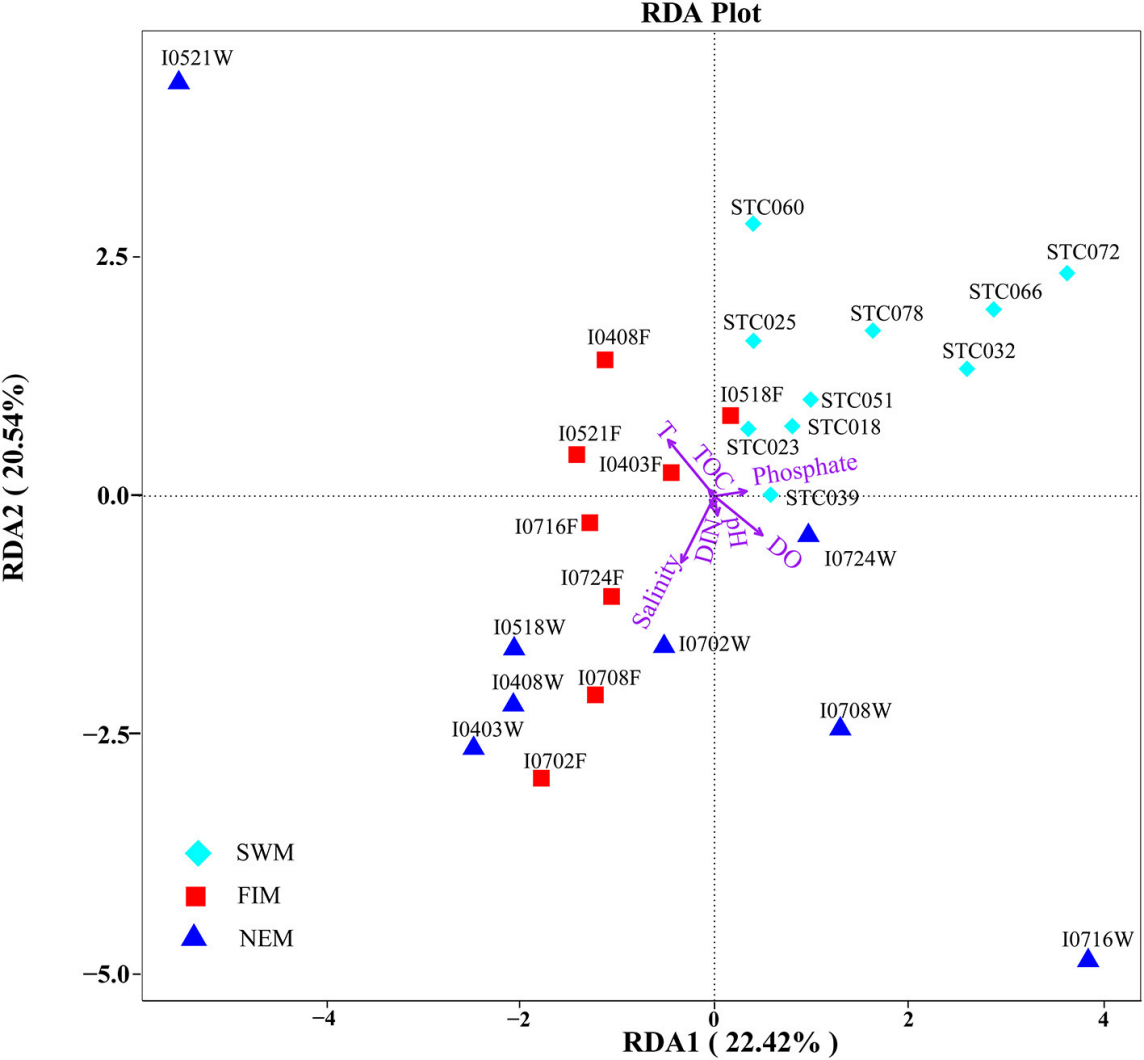
Redundancy discriminant analysis (RDA) of the relationship between bacterial communities and the environmental variables in surface water from the Southwest monsoon (SWM), the fall inter-monsoon (FIM) and the Northeast monsoon (NEM). Correlations between environmental variables (temperature, salinity, pH, DO, phosphate, DIN, TOC) and RDA axes were represented by the length and angle of arrows (environmental factors).

RDA confirmed the first and second axes explained 42.96% of the cumulative variances of the surface bacterial community and environmental factors at the species level (Figure 6). Moreover, the *p*-value displayed the significance of the relationship between the individual environmental factor and bacterial communities, and the values were 0.0015, 0.0005, 0.006, and > 0.32 for temperature, salinity, DO, and other parameters, respectively. All these suggested that temperature, salinity and DO were main factors influencing the bacterial communities (Supplementary Table 5). In addition, we also found salinity had a significant impact on the bacterial community during the fall inter-monsoon (*p* = 0.033), whereas phosphate was the most significant environmental factor during the Southwest monsoon (*p* = 0.01) and the Northeast monsoon (*p* = 0.05) (Supplementary Table 5). Although the water column was strongly stratified, it is interesting to note that none of those environmental parameters significantly affect the bacterial community in the upper water during the Northeast monsoon (*p* > 0.05) (Supplementary Figure 3, Supplementary Table 5).

Spearman's analysis was performed to explore the relationship between bacterial diversity and biogeochemical factors (Supplementary Table 6). It uncovered that the Shannon index of bacteria in surface water was positively associated with temperature (*r* = 0.38, *p* < 0.05) during the Southwest monsoon, while negatively during the fall inter-monsoon (*r* = −0.704, *p* < 0.01). Additionally, salinity and DIN correlated negatively with the diversity of bacteria during the Southwest monsoon and the fall inter-monsoon, respectively, while TOC positively influenced the bacterial diversity during the Northeast monsoon. In the upper water, there was no significant influence on bacterial diversity by any of measured environmental factors, however, it is worth noting that bacterial richness was strongly positively correlated with temperature, DO and phosphate (Supplementary Table 6).

**Figure 7:**
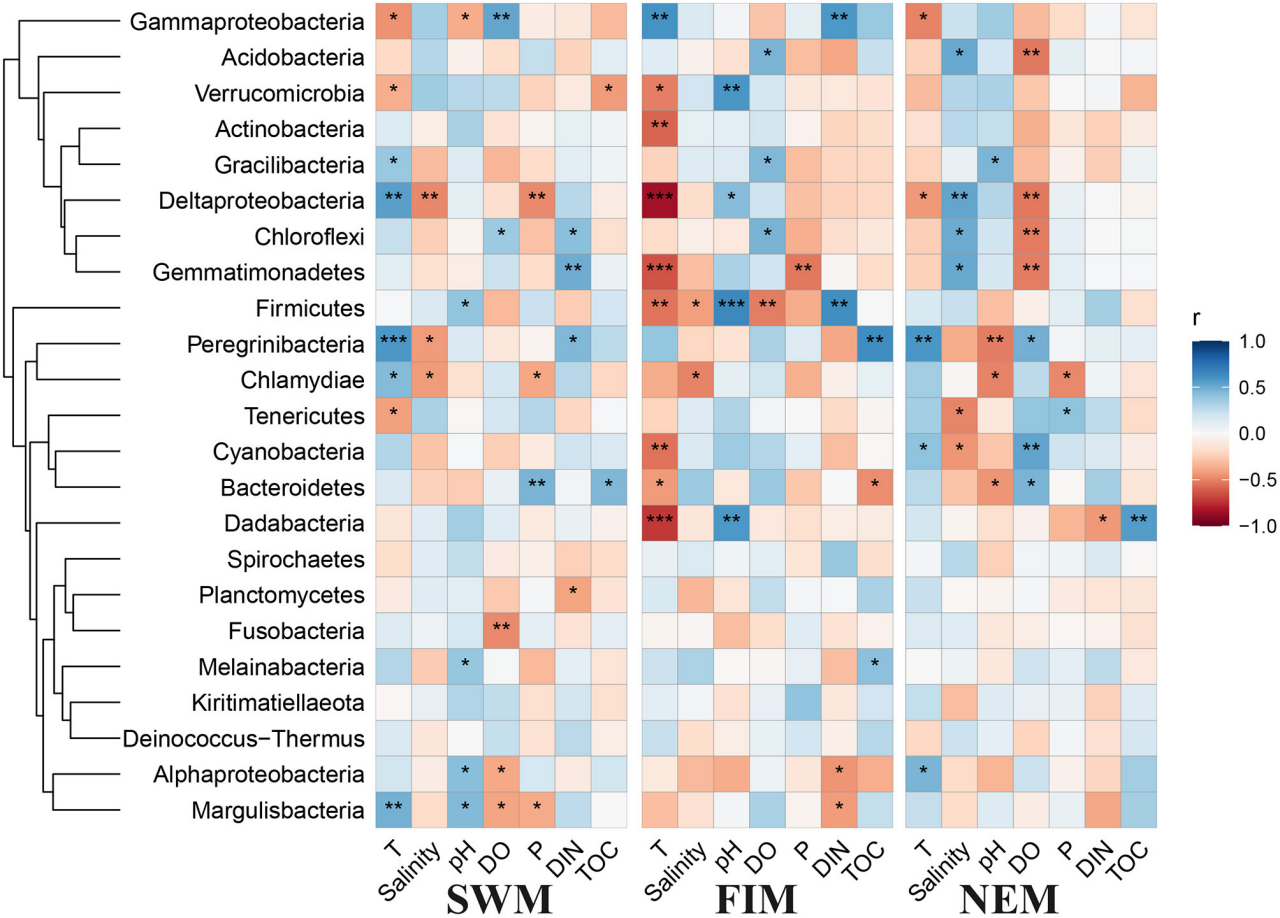
Pearson's correlation analysis between environmental factors and different taxa in surface water during the Southwest monsoon (SWM), the fall transition (FIM), and the Northeast monsoon (NEM), *p < 0.05, **p < 0.01, and ***p < 0.001.

The Pearson's correlation analysis between biogeochemical parameters and the abundance of bacterial taxa in surface water are shown in Figure 7. Among all the environmental variables, temperature, salinity, and DO were the most important factors that strongly influenced the abundance of bacterial taxa. Temperature in particular was significantly positively associated with some of the bacterial taxa during the Southwest monsoon, such as Deltaproteobacteria, Margulisbacteria, and Candidatus Peregrinibacteria, while strongly negatively correlated with Cyanobacteria, Actinobacteria, Firmicutes Dadabacteria, and Gemmatimonadetes during the fall inter-monsoon. Gammaproteobacteria, the dominant class, was significantly positive correlated with temperature during the fall inter-monsoon, yet strongly negative during the Southwest monsoon and the Northeast monsoon. Interestingly, environmental factors have no effect on most of the bacterial taxa in the upper water during the Northeast monsoon, except for Candidatus Campbellbacteria, which strongly positively involve in depth, phosphate and DIN, while negatively associated with temperature, pH, and DO (Supplementary Figure 4A). In addition, several phyla with low relative abundance, such as Planctomycetes and Nitrospirae, positively correlated with salinity (Supplementary Figure 4A).

### Seasonal Bacterial Co-occurrence Networks

**Figure 8:**
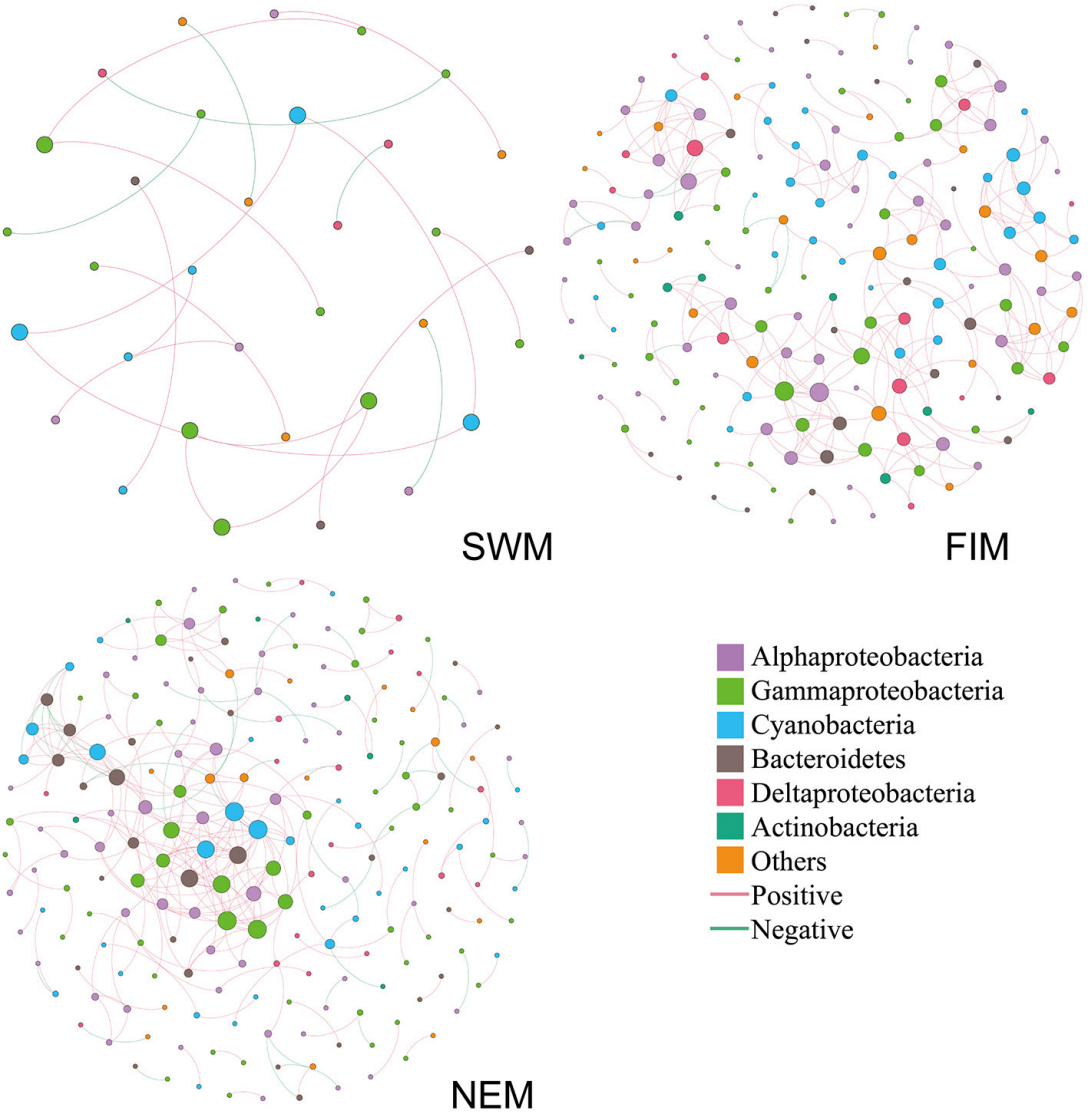
Co-occurrence networks of the bacterial communities during the Southwest monsoon (SWM), the fall inter-monsoon (FIM) and the Northeast monsoon (NEM), respectively, based on pairwise Spearman's correlations between OTUs. The color in the nodes represents the taxa. The size of each node is proportional to the number of connections. A red edge indicates a positive interaction between two individual nodes, while a green edge indicates a negative interaction.

Inter-taxa relationship networks of surface bacteria among seasons were constructed by MENAP based on OTUs (Figure 8). Nodes (OTUs) and edges are fundamental components of a network, representing taxa and correlations, respectively. The network, which only showed strong associations between OTUs, was comprised of 33 nodes and 20 edges during the Southwest monsoon; 188 nodes and 319 edges during the fall inter-monsoon; and 208 nodes and 275 edges during the Northeast monsoon. Bacterial families from Proteobacteria, Cyanobacteria, Bacteroidetes, and Actinobacteria displayed extensive correlations with others. Proteobacteria (Alphaproteobacteria, Gammaproteobacteria, and Deltaproteobacteria) made up most of nodes in the network, especially during the Northeast monsoon, followed by the fall inter-monsoon. Cyanobacteria also attributed to a large portion of nodes in the network. However, compared to the fall inter-monsoon and the Northeast monsoon, there were significantly fewer OTU nodes showing connections with one another during the Southwest monsoon. In addition, it was interesting to note that there were 15, 306 and 231 positive correlations during the Southwest monsoon, the fall inter-monsoon, and the Northeast monsoon, respectively, and correspondingly only 5, 13, and 44 negative correlations. This indicated that the bacterial relationships varied between seasons, and the OTUs were more connected during the fall inter-monsoon, followed by the Northeast monsoon and the Southwest monsoon. The positive correlation was far greater than the negative correlation in the network.

There were only few common OTUs with strong association in all three seasons (Supplementary Figure 5), including Cyanobacteria, Gammaproteobacteria, and Alphaproteobacteria. In general, about half of the OTUs with strong associations were shown up only in one season. During the transition from Southwest monsoon to fall inter-monsoon, most of OTUs with strong association were retained. However, more OTUs with strong association arose during fall inter-monsoon, and about half of those OTUs were found after the transition to Northeast monsoon. The co-occurrence of species within the network could be due to the shared niches, or similar preferences for environmental conditions. Furthermore, it may also indicate that OTU nodes could be more inclined to co-occur with one another (presented as positive correlations) than to be mutually exclusive (presented as negative correlations) in different seasons.

### Predicted Metabolic Potential

**Figure 9:**
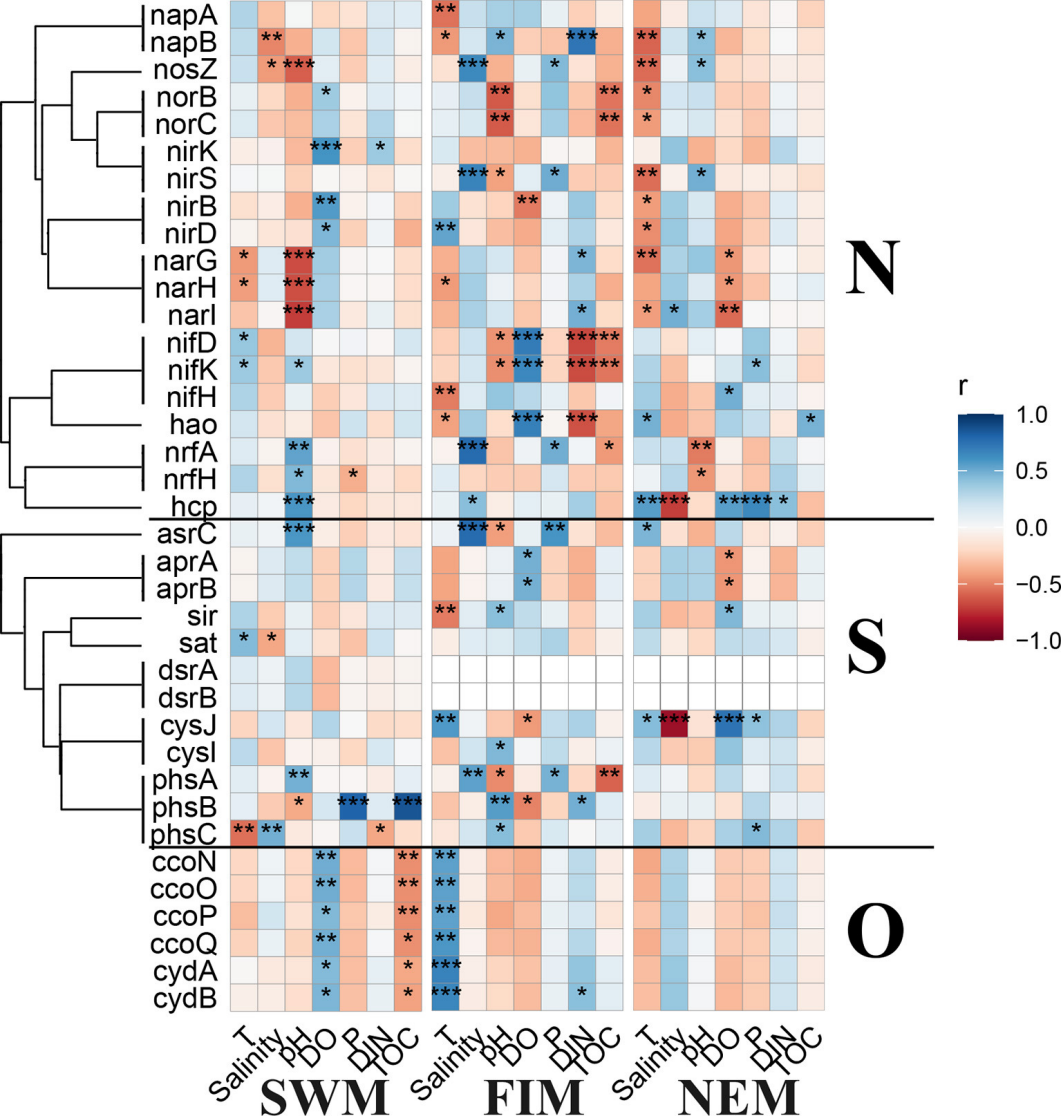
Pearson's correlation analysis between the predicted key functional marker genes related to nitrogen (N), sulfur (S), and oxygen (O) cycling and environmental factors during the Southwest monsoon (SWM), the fall inter-monsoon (FIM) and the Northeast monsoon (NEM), respectively. *p < 0.05, **p < 0.01, and ***p < 0.001.

Predicted metabolisms for elements cycling, e.g., N, S, and O, based on 16S rDNA sequences of the surface bacterial assemblages among seasons and in upper water during the Northeast monsoon demonstrated similar patterns (Supplementary Figure 6). The significant correlations between environmental variables and the abundance of N, S, and O cycling genes were conducted by Pearson's analysis (Figure 9). During the Southwest monsoon, several genes related with N and S cycling had close relationships with pH, and all the O cycling genes showed opposite correlations with DO and TOC. For the fall inter-monsoon, there was more statistically significant correlations between predicted N and S cycling genes with environmental factors than other seasons. During the Northeast monsoon, fewer metabolic genes were extremely influenced by physicochemical factors especially for O cycling genes, which had no remarkable associations with any parameters. With regards to the upper water during the Northeast monsoon, there are fewer statistically significant correlations between the predicted N, S, and O cycling genes with environmental variables when compared with surface water samples (Supplementary Figure 4B).

## Discussion

### Spatial and Seasonal Heterogeneity in Bacterial Community Structure

Extensive analyses of bacterial diversity and richness were carried out in the Eastern Tropical Indian Ocean, covering three seasons and a high spatial range from 4°N to 8°S. The bacterial richness in this area was much lower than that of the Pacific Ocean, both in summer and winter (Suh et al., 2014). A possible reason for this is the suppression on up-welling and mixing of the deep waters, the Eastern Equatorial Indian Ocean is made up of a typical oligotrophic area (Kumar et al., 2009). On a seasonal scale, the diversity and richness in surface water during the Northeast monsoon (winter) were significantly higher than those during the Southwest monsoon (summer), which was consistent with a similar study in the Pacific Ocean (Suh et al., 2014). According to the LEfSe analysis (Figure 5), Cyanobacteria are higher during the Northeast monsoon than during the Southwest monsoon and the fall inter-monsoon, though there was no significant difference among seasons. The equatorial region has plenty of sunshine and high temperature all year round and only exhibits a weak seasonal variation, while, most of sampling sites during this study located south of the equator. The light availability and temperature in the sampling sites are increasing during the period when the sun is moving from the Northern Hemisphere to the Southern Hemisphere, i.e., from the Southwest monsoon to the fall inter-monsoon, and to the Northeast monsoon. Thus, the highest abundance of Cyanobacteria during the Northeast monsoon may due to the combined effect of relative high temperature and strong sunshine in the sampling sites during the Northeast monsoon, but this tendency still did not show any significant differences among seasons. The marine bacterial diversity has been reported to peak at high latitudes in winter (Ladau et al., 2013), in contrast to the seasonally consistent high diversity of macro-organisms (Pommier et al., 2007). In the equatorial area, the microbial communities in the Singapore Strait showed a seasonal variability which might be caused by different monsoon (Chénard et al., 2019). The bacterial diversity and richness in our study were higher at high latitudes (north of the equator) than low latitudes (south of the equator) during the fall inter-moon (fall), but not during the Northeast monsoon (winter).

Microbial communities could be easily affected by the physicochemical conditions, e.g., currents or precipitation, and the influence is more remarkable for the surface water. During different monsoon seasons, the shift of microbial community in the Singapore Strait, located in the equatorial area, was mainly due to the seasonal reversal ocean currents (Chénard et al., 2019). The Wyrtki Jet, which occurs annually in the Indian Ocean monsoon transition period, and carries high salinity water from the Arabian Sea to the east, by advection along the equator to change the environmental conditions on both sides of the equator (Wyrtki, 1973), may contribute to the difference of microbial communities between north and south of the equator. For example, Alteromonadaceae, one of the most abundant families, was detected in much higher abundance south of the equator (32.06%) than north of the equator (8.28%) during the fall inter-monsoon; however, Actinomarinaceae, Synechococcales, and Cyanobacteria, all demonstrated a trend in the opposite direction. The relative abundance of Cyanobacteria (mainly *Prochlorococcus* genus) have been reported higher in the tropical latitudes than in the high latitudes in the Pacific Ocean, particularly during summer (Suh et al., 2014), which is different from our observation in the Eastern Tropical Indian Ocean.

During the Northeast monsoon, the composition of bacterial community changed along depth in the upper water. The abundance of Gammaproteobacteria increased with depth, which was consistent with the previous studies in the North Atlantic (Lauro and Bartlett, 2008; AgoguÉ et al., 2011). The Eastern Equatorial Indian Ocean is a typical oligotrophic area (Kumar et al., 2009), and Gammaproteobacteria have been reported to adopt to oligotrophic environments with tolerance to a wide temperature range (Cho and Giovannoni, 2004). Seasonal monsoons induce strong stratification between 75 and 150 m in the Indian Ocean (Fine et al., 2008), with the density difference between waters above and below the thermocline creating a barrier to vertical mixing. This has enabled the bacterial community composition at different water layers to diverge in the upper waters.

### Correlation Between Bacterial Taxa and Environmental Parameters

Biogeochemical characteristics, including temperature, nutrients, salinity, and DO, are the important regulators of bacterial community composition in many marine ecosystems (Biddanda et al., 2001; Naqvi, 2006; Paulmier and Ruiz-Pino, 2009; Guo et al., 2018; Gao et al., 2020). The temperature of surface water in the Indian Ocean is closely related to the seasonal variation of the monsoon. The area with high temperature during the Southwest monsoon is distributed in the Northeast tropical Indian Ocean, while the low surface temperature is always distributed in the southern tropical Indian Ocean. The area with high surface temperature is located near the equator during the Northeast monsoon. In general, the surface seawater temperature in the studied area has small variations in different seasons, but it is still an important factor affecting the bacterial community distribution based on our observations. With respective to the salinity in surface water in different seasons, the influence of zonal advection driven by monsoon, the increased salinity due to the high rate of evaporation during the Southwest monsoon, and freshwater forcing during the Northeast monsoon are the dominant mechanisms of sea surface salinity variability (Nyadjro et al., 2011). Our data indicate that salinity is one of the most significant environmental parameters that regulated bacterial community structures during the fall inter-monsoon. This may be explained by the Wyrtki Jet develops during the inter-monsoon that advects an equatorial tongue of water across the basin, and reinforces the east-west salinity gradient (Masson et al., 2002). Additionally, Martiny et al. (2011) hypothesized that geographical distance was also a key factor driving microbial distribution patterns in sediments, and (Liu et al., 2014) concluded latitude might be the crucial variable responsible for the observed microbial community patterns. However, the overall extent of horizontal variation on bacterial community structure was small among seasons in this area, except for the fall inter-monsoon, which was strongly affected by Wyrtki Jet. Therefore, based on our current observation, the small differences of bacterial communities among the study sites in the horizontal level indicated that the relative homogeneous bacterial distribution in the size of our sampling scale.

Pearson's correlation analysis revealed that most environmental parameters, especially temperature, salinity, DO, pH, and DIN, have close associations with some bacterial taxa in different seasons. Temperature for example, has significantly positive/negative correlations with Gammaproteobacteria and Deltaproteobacteria during the three seasons. This indicates that the effect of individual environmental parameters on bacterial distribution differs among seasonal scales.

With respect to vertical gradients in the upper water during the Northeast monsoon, temperature, pH, DO, and nutrients varied little in the uniform mixing layer, but changed sharply in the thermocline (Supplementary Table 1). The concentration of DO reached the maximum at 75 m and decreased with the increase of depth. The possible reason was that the maximum chlorophyll layer was at about 75 m, and photosynthetic bacteria released a large amount of oxygen in this area. In addition, it is interesting to note that none of the above-mentioned environmental parameters showed any statistically significance with bacterial community composition (Supplementary Table 5), although strong stratification along 100–150 m caused significant changes to environmental parameters. At the same time, only three bacteria taxa in upper waters during the Northeast monsoon had significant association with environmental factors according to Pearson's correlation analysis (Supplementary Figure 4A). Taken together, these findings imply that the overall extent of environmental factors on seasonal community variations is bigger than that in vertical variation.

### Co-occurrence Network: Seasonality With Bacterial Community Structure

Interactions among bacterial communities could be partially explored by co-occurrence networks (Faust and Raes, 2012). Previous studies have attempted to use networks to reveal potential couplings among bacterial taxa in different seasons (Chafee et al., 2018; Liu et al., 2020). All of these studies found a clear seasonal pattern of correlations, with bacterial assembly possessing lower interconnected networks in summer compared with winter. One explanation may be attributed to the lower summer spatial connectivity, which was driven by the strong Southwest monsoon, and the monsoon-driven circulation such as the Southwest Monsoon Current and the South Equatorial Countercurrent (Schott and McCreary, 2001). These may result in more scattered co-occurrence pattern of bacteria in summer. The rainfalls were strongest during the Northeast monsoon which may affect the bacterial associations by changing the salinity or concentration of nutrients within micro-environments. However, more than half of the OTUs with strong associations during the Northeast monsoon were retained from fall inter-monsoon.

In our findings, the co-occurrence network analysis in surface seawater samples displayed that positive co-occurrences accounted for up to 90% of all associations, which was in agreement with the observations in the previous studies (Lima-Mendez et al., 2015; Milici et al., 2016) (Figure 8). This is probably due to the similar environmental preference and/or the high resistance of marine prokaryotes to environmental stresses, which enable them to coexist in the same ecological niche (Liu et al., 2019).

### Predicted Metabolic Potentials

Marine microbes play a big part in diverse biochemical processes, including carbon cycling (Kirchman, 2015), nitrogen fixation and removal (Barlett and Leff, 2010; Qian et al., 2018) and sulfate reduction (Guo et al., 2018). Based on the 16S rDNA sequencing data, functional genes in the samples were predicted. In the Eastern Tropical Indian Ocean, the relative abundance of predicted key functional genes related to N, S, and O cycling from surface samples in different seasons, and from the upper water during the Northeast monsoon showed slight variation among them (Supplementary Figure 6). Predicted genes encoding for molybdenum-iron nitrogenase (MoFe, *nifDHK*) for fixation of N_2_ and associated with Proteobacteria, Bacteroidetes, Cyanobacteria, Firmicutes, Chlorobi, and Verrucomicrobia (He et al., 2020) were detected to be closely related to more environmental factors during the fall inter-monsoon. The abundance of the predicted *nifH* gene was higher than the other genes related to nitrogen cycling, suggesting that the bacterial fixation of N_2_ is more frequent in the upper waters ( ≤ 300 m) (Supplementary Figure 6). In addition, predicted *nrfH* gene encoding for nitrite reductase (cytochrome; ammonia-forming) was detected with slightly higher abundance during the Southwest monsoon than in the Northeast monsoon and the fall inter-monsoon, indicating that the denitrification process was more common during the Southwest monsoon.

Sulfur-oxidizing bacteria are particularly abundant at the oxic-anoxic interfaces, where O_2_, NO^3−^, and metal oxides are available as electron acceptors (Callbeck et al., 2018; He et al., 2020). At these interfaces, H_2_S can be oxidized using sulfide to form ${\text{SO}}_{3}^{2-}$. ${\text{SO}}_{3}^{2-}$ can be further oxidized to ${\text{SO}}_{4}^{2-}$ by adenylylsulfate reductase (*apr*) and sulfate adenylyltransferase (*sat*) (Wasmund et al., 2017). In this study, predicted genes encoding for adenylylsulfate reductase (*aprAB*) and sulfate adenylyltransferase (*sat*) were enriched, both in surface water in different seasons and in upper water during the Northeast monsoon (Supplementary Figure 6). Additionally, some predicted genes involved in the dissimilatory sulfate reduction process were found in relatively low abundances, for example, *dsrAB*. Taken together, a possible reason is that the oxygen in the upper water was sufficient and the sulfur oxidation process was more frequent, while the dissimilatory sulfur reduction process was less common.

In terms of oxygen cycling, predicted genes encoding for two types of terminal oxidase: *cbb*_3_-type cytochrome *c* oxidase (*ccoNOPQ*) (Cosseau and Batut, 2004) and cytochrome *bd* type oxidase (*cydAB*) (Morris and Schmidt, 2013) with high affinities for O_2_ were found with high abundance in all the samples, which indicate that aerobic respiration is predominant in upper waters. Interestingly, all the terminal oxidases with high affinities for oxygen showed significantly positive correlations with DO only during the Southwest monsoon, but had significantly negative correlations with TOC (Figure 9). This indicates organic matter decomposition mainly depends on aerobic respiration, using oxygen as the electron acceptor instead of alternative electron acceptors, such as nitrate and nitrite. Nevertheless, high-affinity terminal oxidases are considered to be dominant under low oxygen conditions (Morris and Schmidt, 2013), especially when oxygen is the limited substrate for aerobic respiration (Gong et al., 2016). However, the oxygen concentrations during this study (over 200 μmol/L) were much higher than the normally considered low oxygen conditions (few micromolar or even nanomolar level oxygen). The regulation of gene expressions of different types of terminal oxidases is hardly only attributed to oxygen concentrations (Gong et al., 2018).

## Conclusion

This study demonstrated that the bacterioplankton community of the Eastern Tropical Indian Ocean consisted of various taxa that were commonly detected in marine environments, such as Proteobacteria, Cyanobacteria, Actinobacteria, Bacteroidetes, and Chloroflexi. The bacterial community composition significantly correlated with some environmental variables that influenced the bacterial community, such as temperature, salinity, and DO in surface water. The bacterial community composition showed strong seasonality, as well as vertical variations; however, the impact of environmental factors on seasonal community variations was greater than that of vertical variation. Seasonal bacterial co-occurrence networks indicated that the bacterial relationship with one another varied among seasons, and the networks in surface water during the Southwest monsoon were much less complex than those during the fall inter-monsoon and the Northeast monsoon. Moreover, there was a greater statistically significant linear correlation between the abundance of N, S, and O cycling genes with environmental variables in surface water at different seasons than in the upper water during the Northeast monsoon, although strong thermal stratification was detected in the upper water in the Eastern Tropical Indian Ocean. We also found that genes related to N and S cycling demonstrated closer associations with environmental factors during the fall inter-monsoon, whereas terminal oxidases with high affinities for oxygen showed opposite correlations with DO and TOC during the Southwest monsoon. This study provided a detailed survey on the bacterial community structure and function, in response to changes in seasonal as well as vertical variations in the Eastern Tropical Indian Ocean.

## Data Availability Statement

The datasets presented in this study can be found in online repositories. The names of the repository/repositories and accession number(s) can be found at: https://www.ncbi.nlm.nih.gov/, SRR12474819.

## Author Contributions

PG and GD collected samples. QW and XZ performed the measurement of environmental characteristics. PG and DZ analyzed the data. PG and LQ drafted the manuscript. LQ and XG conceived and designed the study, and revised the manuscript. All authors approved the manuscript.

## Conflict of Interest

The authors declare that the research was conducted in the absence of any commercial or financial relationships that could be construed as a potential conflict of interest.
