# Supplementary material for: Influences of Seasonal Monsoons on the Taxonomic Composition and Diversity of Bacterial Community in the Eastern Tropical Indian Ocean
Source: Front Microbiol. 2021 Jan 26;11:615221. doi: 10.3389/fmicb.2020.615221 (PMC7870504; doi:10.3389/fmicb.2020.615221)
Supplement: Supplementary file 1 [file Data_Sheet_1.docx]

**TABLE S1.** Physico-chemical characteristics of surface water during the SWM, the FIM and the NEM, respectively and in upper waters (3 -300 m with six depths) during the NEM in the Eastern Tropical Indian Ocean. SWM, Southwest monsoon; FIM, fall inter-monsoon; NEM, Northeast monsoon.

| **Sampling station** | **Coordinates** | **Depths**  **(m)** | **Temperature**  **(°C)** | **Salinity**  **(PSU)** | **pH** | **DO**  **(µmol/L)** | **Phosphate**  **(****µmol/L)** | **DIN (µmol/L)** | **TOC**  **(****mg/L)** |
| --- | --- | --- | --- | --- | --- | --- | --- | --- | --- |
| **SWM** |  |  |  |  |  |  |  |  |  |
| STC018 | 092°59.983′E  0°05.010′S | 3 | 29.57 | 33.92 | 8.12 | 207.904 | 0.054 | 2.059 | 1.410 |
| STC023 | 093°28.888′E  2°02.101′S | 3 | 29.77 | 33.66 | 8.08 | 202.799 | 0.023 | 1.179 | 0.957 |
| STC025 | 088°00.312′E  4°59.647′S | 3 | 29.63 | 34.28 | 8.15 | 193.258 | 0.130 | 0.867 | 2.070 |
| STC032 | 091°09.047′E  5°00.116′S | 3 | 28.98 | 34.06 | 8.09 | 204.283 | 0.300 | 1.119 | 3.830 |
| STC039 | 094°17.970′E  5°00.196′S | 3 | 29.57 | 33.46 | 8.10 | 205.146 | 0.102 | 2.416 | 2.840 |
| STC051 | 088°29.875′E  8°00.032′S | 3 | 27.38 | 34.80 | 8.10 | 205.585 | 0.155 | 0.998 | 1.310 |
| STC060 | 093°00.000′E  8°00.072′S | 3 | 28.28 | 34.72 | 8.10 | 204.460 | 0.071 | 1.194 | 1.490 |
| STC066 | 095°59.972′E  8°00.115′S | 3 | 27.88 | 34.70 | 8.13 | 201.552 | 0.124 | 0.340 | 0.861 |
| STC072 | 099°00.203′E  8°00.140′S | 3 | 28.81 | 34.30 | 8.15 | 204.215 | 0.101 | 1.009 | 1.300 |
| STC078 | 102°00.109′E  8°00.079′S | 3 | 29.44 | 34.05 | 8.18 | 202.208 | 0.051 | 1.188 | 1.440 |
| **FIM/NEM** |  |  |  |  |  |  |  |  |  |
| I0403F | 092°30.053′E  00°00.031′ N | 3  3 | 28.59 | 34.35 | 8.24 | 218.929 | 0.069 | 0.923 | 1.085 |
| I0403W |  |  | 29.31 | 33.72 | 8.23 | 215.283 | 0.547 | 2.443 | 1.260 |
| I0408F | 089°42.551′ E  00°00.101′N | 3  3 | 28.74 | 35.58 | 8.22 | 212.102 | 0.243 | 1.137 | 0.544 |
| I0408W |  |  | 29.36 | 34.11 | 8.22 | 214.429 | 0.065 | 0.67 | 0.720 |
| I0518F | 095°39.050′ E  05°00.072′S | 3  3 | 29.01 | 33.82 | 8.24 | 212.253 | 0.202 | 1.306 | 4.349 |
| I0518W |  |  | 29.10 | 34.63 | 8.22 | 213.453 | 0.278 | 2.241 | 0.921 |
| I0521F | 097°00.060′ E  05°00.065′S | 3  3 | 28.87 | 33.84 | 8.23 | 207.167 | 0.161 | 1.333 | 0.727 |
| I0521W |  |  | 29.12 | 34.86 | 8.21 | 207.987 | 0.150 | 1.921 | 2.869 |
| I0702F | 087°59.874′ E  03°59.922′N | 3  3 | 28.34 | 33.29 | 8.27 | 206.407 | 0.080 | 1.809 | 1.929 |
| I0702W |  |  | 29.23 | 34.51 | 8.22 | 209.910 | 0.260 | 0.582 | 5.955 |
| I0708F | 087°59.813′ E  00°59.990′N | 3  3 | 28.57 | 34.89 | 8.28 | 207.087 | 0.197 | 1.308 | 2.409 |
| I0708W |  |  | 28.96 | 34.68 | 8.28 | 204.523 | 0.254 | 1.074 | 2.522 |
| I0716F | 088°00.008′ E  02°59.965′S | 3  3 | 29.07 | 34.48 | 8.24 | 208.905 | 0.143 | 1.365 | 5.049 |
| I0716W |  |  | 29.02 | 35.29 | 8.24 | 204.038 | 0.313 | 1.637 | 2.181 |
| I0724F | 087°59.929′ E  06°59.977′S | 3  3 | 28.84 | 34.87 | 8.25 | 212.749 | 0.115 | 1.992 | 0.800 |
| I0724W |  |  | 28.78 | 34.46 | 8.27 | 209.239 | 0.233 | 1.377 | 0.700 |
| **NEM** |  |  |  |  |  |  |  |  |  |
| I710S | 087°59.758′E  00°00.155′S | 3  30  75  100  150  300 | 29.07 | 34.71 | 8.20 | 208.217 | 0.237 | 0.906 | 2.846 |
| I71030 |  |  | 29.11 | 34.85 | 8.22 | 206.692 | 0.211 | 0.942 | 5.097 |
| I71075 |  |  | 28.72 | 35.58 | 8.20 | 219.865 | 0.226 | 4.254 | 3.644 |
| I710100 |  |  | 28.14 | 35.55 | 8.18 | 190.491 | 0.358 | 3.055 | 5.915 |
| I710150 |  |  | 13.87 | 35.11 | 7.77 | 66.518 | 1.917 | 29.379 | 3.688 |
| I710300 |  |  | 11.36 | 35.03 | 7.73 | 75.103 | 2.127 | 33.704 | 5.474 |
| BUOYS | 099°59.863′E  08°01.490′N | 3  30  75  100  150  300 | 28.17 | 34.20 | 8.21 | 211.079 | 0.309 | 0.855 | 0.445 |
| BUOY30 |  |  | 27.89 | 34.19 | 8.30 | 209.456 | 0.351 | 1.253 | 1.601 |
| BUOY75 |  |  | 22.75 | 34.50 | 8.26 | 227.787 | 0.384 | 0.879 | 0.751 |
| BUOY100 |  |  | 18.60 | 34.51 | 8.25 | 194.732 | 0.469 | 3.031 | 0.668 |
| BUOY150 |  |  | 15.37 | 34.86 | 8.00 | 117.673 | 1.420 | 24.443 | 0.613 |
| BUOY300 |  |  | 10.91 | 34.87 | 7.85 | 100.279 | 1.485 | 33.556 | 0.397 |

**TABLE S2.** Diversity and richness of bacteria in surface water during the Southwest monsoon (SWM), the fall inter-monsoon (FIM) and the Northeast monsoon (NEM), respectively and in upper waters (3 -300 m with six depths) during the Northeast monsoon (NEM) in the Eastern Tropical Indian Ocean.

| **Time** | **Station** | **Depth** | **Raw reads** | **Clean reads** | **Filtered**  **reads** | **OTUs** | **Shannon** | **Chao1** |  |
| --- | --- | --- | --- | --- | --- | --- | --- | --- | --- |
| **SW** |  |  |  |  |  |  |  |  |  |
|  | STC018 | 3 | 79499 | 75631 | 3868 | 637 | 6.05 | 797.15 |  |
|  | STC023 | 3 | 83952 | 80234 | 3718 | 588 | 5.99 | 705.27 |  |
|  | STC025 | 3 | 81958 | 78851 | 3107 | 588 | 5.77 | 716.81 |  |
|  | STC032 | 3 | 89639 | 85483 | 4156 | 647 | 6.13 | 807.66 |  |
|  | STC039 | 3 | 86452 | 81026 | 5426 | 605 | 5.95 | 713.79 |  |
|  | STC051 | 3 | 88638 | 82917 | 5720 | 620 | 5.57 | 739.24 |  |
|  | STC060 | 3 | 85728 | 80158 | 5570 | 596 | 5.44 | 693.30 |  |
|  | STC066 | 3 | 84048 | 80266 | 3782 | 621 | 5.77 | 727.57 |  |
|  | STC072 | 3 | 84508 | 80097 | 4411 | 616 | 5.83 | 707.45 |  |
|  | STC078 | 3 | 83663 | 80210 | 3453 | 676 | 6.17 | 799.37 |  |
| **FIM** |  |  |  |  |  |  |  |  |  |
|  | I0403F | 3 | 86600 | 80111 | 6489 | 681 | 6.41 | 863.11 |  |
|  | I0408F | 3 | 86950 | 80110 | 6840 | 616 | 6.34 | 754.63 |  |
|  | I0518F | 3 | 84153 | 79589 | 4564 | 540 | 5.54 | 697.78 |  |
|  | I0521F | 3 | 88922 | 84715 | 4207 | 407 | 4.70 | 509.58 |  |
|  | I0702F | 3 | 85805 | 82401 | 3404 | 693 | 6.34 | 894.87 |  |
|  | I0708F | 3 | 85717 | 80249 | 5468 | 688 | 6.27 | 840.76 |  |
|  | I0716F | 3 | 80566 | 77482 | 3084 | 502 | 5.47 | 606.78 |  |
|  | I0724F | 3 | 88952 | 85456 | 3497 | 536 | 5.51 | 664.31 |  |
| **NEM** |  |  |  |  |  |  |  |  |  |
|  | I0403W | 3 | 85701 | 80204 | 5497 | 795 | 6.26 | 877.81 |  |
|  | I0408W | 3 | 85392 | 80164 | 5228 | 877 | 6.25 | 927.19 |  |
|  | I0518W | 3 | 84390 | 80111 | 4279 | 806 | 6.15 | 870.04 |  |
|  | I0521W | 3 | 83388 | 80165 | 3223 | 780 | 6.63 | 876.64 |  |
|  | I0702W | 3 | 86076 | 80192 | 5885 | 855 | 6.36 | 924.77 |  |
|  | I0708W | 3 | 84111 | 80420 | 3691 | 877 | 6.21 | 962.72 |  |
|  | I0716W | 3 | 84309 | 80117 | 4193 | 1023 | 6.73 | 1099.40 |  |
|  | I0724W | 3 | 83284 | 80109 | 3175 | 783 | 5.55 | 822.61 |  |
| **NEM** |  |  |  |  |  |  |  |  |  |
|  | I710S | 3 | 84847 | 82408 | 2440 | 901 | 6.52 | 876.08 |  |
|  | I71030 | 30 | 83770 | 80152 | 3618 | 864 | 5.80 | 853.03 |  |
|  | I71075 | 75 | 84619 | 80099 | 4521 | 867 | 6.89 | 850.88 |  |
|  | I710100 | 100 | 83817 | 80176 | 3641 | 991 | 6.63 | 967.35 |  |
|  | I710150 | 150 | 82910 | 80186 | 2724 | 1277 | 6.86 | 1256.48 |  |
|  | I710300 | 300 | 84498 | 80155 | 4342 | 927 | 5.40 | 1013.18 |  |
|  | BUOYS | 3 | 84403 | 80111 | 4292 | 857 | 6.69 | 853.83 |  |
|  | BUOY30 | 30 | 85393 | 80156 | 5238 | 937 | 6.53 | 900.19 |  |
|  | BUOY75 | 75 | 86489 | 80123 | 6366 | 876 | 6.42 | 856.67 |  |
|  | BUOY100 | 100 | 83932 | 79486 | 4445 | 944 | 5.77 | 923.54 |  |
|  | BUOY150 | 150 | 84854 | 80184 | 4670 | 913 | 5.49 | 896.51 |  |
|  | BUOY300 | 300 | 85536 | 80180 | 5356 | 1035 | 6.24 | 1009.36 |  |

**TABLE** **S3.** Wilcox test of the statistical differences among the groups (within seasons, water layers) of Alpha diversity (Shannon and Chao1).

| **Group-Pair** | **Difference** | | ***p* value** | |
| --- | --- | --- | --- | --- |
|  | **Shannon** | **Chao1** | **Shannon** | **Chao1** |
| NEM-SWM | 19.5417 | 33.9250 | 0.0013^**^ | 0^**^ |
| NEM-FIM | 15.5417 | 32.2083 | 0.0122^*^ | 0^**^ |
| I710 150 m-I710 30 m | 9.6667 | 9.6667 | 0.0278^*^ | 0.0383^*^ |
| I710 30 m-I710 75 m | -10.3333 | -1.0000 | 0.0202^*^ | 0.8138 |
| BUOY150 m-BUOY75 m | -8.6667 | 3.3333 | 0.0450^*^ | 0.5104 |
| BUOY150 m-BUOYS m | -9.6667 | 2.3333 | 0.0281^*^ | 0.6434 |

SWM, Southwest monsoon; FIM, fall inter-monsoon; NEM, Northeast monsoon.

* *p* < 0.05, ** *p* < 0.01.

**TABLE** **S4.** Statistical differences of community structure among groups (within locations, water layers) were conducted by ANOSIM test.

| **Group-Pair** | R | *p* |
| --- | --- | --- |
| Samples from North / South of the equator during the FIM | 0.788 | 0.001^**^ |
| Samples from 3 -75 m/  100 -300 m during the NEM | 0.208 | 0.002^**^ |

Fall inter-monsoon (FIM); Northeast monsoon (NEM)

* *p* < 0.05, ** *p* < 0.01.

**TABLE S5. RDA's rank order correlation analysis**

|  | **Depth**  **(m)** | **Temperature**  **(°C)** | **Salinity**  **(PSU)** | **pH** | **DO**  **(µmol/L)** | **Phosphate**  **(µmol/L)** | **DIN**  **(µmol/L)** | **TOC**  **(µmol/L)** |
| --- | --- | --- | --- | --- | --- | --- | --- | --- |
| **SWM** |  |  |  |  |  |  |  |  |
| RDA1 | - | -0.1445 | -0.0136 | -0.1965 | 0.8571 | 0.9786 | 0.9870 | 0.9980 |
| RDA2 | - | 0.9895 | -0.9999 | 0.9805 | -0.5152 | -0.2060 | 0.1607 | -0.0627 |
| R^2^ | - | 0.3005 | 0.2116 | 0.4196 | 0.0323 | 0.7539 | 0.0194 | 0.5711 |
| *p* | - | 0.2659 | 0.4148 | 0.1499 | 0.8411 | 0.0100^**^ | 0.9310 | 0.0610 |
| **FIM** |  |  |  |  |  |  |  |  |
| RDA1 | - | -0.0670 | -0.4261 | 0.9248 | -0.4172 | -0.9708 | -0.6255 | 0.8454 |
| RDA2 | - | 0.9978 | -0.9047 | -0.3806 | -0.9088 | -0.2400 | 0.7802 | 0.5342 |
| R^2^ | - | 0.1049 | 0.7713 | 0.2661 | 0.1368 | 0.1654 | 0.1171 | 0.4203 |
| *p* | - | 0.7391 | 0.0325^*^ | 0.4513 | 0.7186 | 0.6317 | 0.7401 | 0.2554 |
| **NEM** |  |  |  |  |  |  |  |  |
| RDA1 | - | 0.2642 | -0.7703 | 0.9859 | -0.2144 | 0.5759 | -0.0534 | 0.1074 |
| RDA2 | - | 0.9645 | 0.6377 | 0.1675 | -0.9767 | -0.8175 | -0.9986 | 0.9942 |
| R^2^ | - | 0.0082 | 0.1412 | 0.0874 | 0.1910 | 0.7510 | 0.5984 | 0.1944 |
| *p* | - | 0.9755 | 0.6217 | 0.8136 | 0.6552 | 0.0500^*^ | 0.0755 | 0.4893 |
| **Overall** |  |  |  |  |  |  |  |  |
| RDA1 | - | -0.6390 | -0.4529 | 0.1528 | 0.7782 | 0.9883 | -0.2696 | -0.6532 |
| RDA2 | - | 0.7692 | -0.8916 | -0.9883 | -0.6281 | 0.1528 | -0.9630 | 0.7572 |
| R^2^ | - | 0.4783 | 0.4958 | 0.0345 | 0.3437 | 0.0947 | 0.0126 | 0.0071 |
| *p* | - | 0.0015^**^ | 0.0005^**^ | 0.6602 | 0.0060^**^ | 0.3168 | 0.8726 | 0.9070 |
| **Upper water during NEM** |  |  |  |  |  |  |  |  |
| RDA1 | -0.9913 | 0.8586 | 0.0865 | 0.6868 | 0.6708 | -0.6347 | -0.8289 | 0.7258 |
| RDA2 | -0.1319 | 0.5126 | -0.9963 | 0.7268 | 0.7416 | -0.7728 | -0.5594 | -0.6879 |
| R^2^ | 0.3701 | 0.3462 | 0.0215 | 0.3146 | 0.4492 | 0.4106 | 0.4594 | 0.0877 |
| *p* | 0.1374 | 0.1529 | 0.9110 | 0.1879 | 0.0590 | 0.0865 | 0.0515 | 0.6897 |

SWM, Southwest monsoon; FIM, fall inter-monsoon; NEM, Northeast monsoon.

Notes: r: RDA's rank order correlation analysis, *p*: *p*-value.
* *p* < 0.05, ** *p* < 0.01.

**TABLE S6. Spearman’s correlation analysis between the environmental factors and the diversity and richness of bacteria**

|  | **Observedspecies** | | **Shannon** | | **Chao1** | |
| --- | --- | --- | --- | --- | --- | --- |
| **SWM** | *p* | r | *p* | r | *p* | r |
| Temperature | 0.9241 | -0.0182 | 0.0381^*^ | 0.3803 | 0.4083 | -0.1567 |
| Salinity | 0.5896 | -0.1026 | 0.0096^**^ | -0.4654 | 0.5092 | 0.1254 |
| pH | 0.1342 | 0.2798 | 0.8011 | 0.0480 | 0.2035 | 0.2390 |
| DO | 0.7301 | -0.0657 | 0.6699 | -0.0811 | 0.5370 | -0.1173 |
| Phosphate | 0.7864 | 0.0516 | 0.6192 | -0.0945 | 0.0585 | 0.3493 |
| DIN | 0.9748 | 0.0060 | 0.3402 | 0.1804 | 0.2948 | -0.1978 |
| TOC | 0.3683 | 0.1703 | 0.1466 | 0.2716 | 0.1617 | 0.2621 |
| **FIM** |  |  |  |  |  |  |
| Temperature | 0.0000^**^ | -0.8408 | 0.0001^**^ | -0.7040 | 0.0005^**^ | -0.6568 |
| Salinity | 0.9903 | 0.0026 | 0.2715 | 0.2338 | 0.8934 | -0.0289 |
| pH | 0.0132^*^ | 0.4981 | 0.3060 | 0.2180 | 0.0171^*^ | 0.4819 |
| DO | 0.4619 | -0.1577 | 0.9514 | 0.0131 | 0.5822 | -0.1182 |
| Phosphate | 0.1832 | -0.2812 | 0.4250 | -0.1708 | 0.5654 | -0.1235 |
| DIN | 0.2294 | -0.2549 | 0.0408^*^ | -0.4203 | 0.1335 | -0.3152 |
| TOC | 0.7511 | 0.0683 | 0.7143 | -0.0788 | 0.2660 | 0.2364 |
| **NEM** |  |  |  |  |  |  |
| Temperature | 0.6426 | -0.0998 | 0.0817 | 0.3625 | 0.6338 | -0.1025 |
| Salinity | 0.2499 | 0.2443 | 0.1708 | 0.2890 | 0.3828 | 0.1865 |
| pH | 0.3368 | 0.2049 | 0.0683 | -0.3783 | 0.2552 | 0.2417 |
| DO | 0.1301 | -0.3179 | 0.5489 | -0.1287 | 0.1920 | -0.2758 |
| Phosphate | 0.9127 | 0.0236 | 0.6961 | 0.0841 | 0.7143 | 0.0788 |
| DIN | 0.0890 | -0.3546 | 0.9320 | 0.0184 | 0.0996 | -0.3441 |
| TOC | 0.9514 | -0.0131 | 0.0105^*^ | 0.5123 | 0.8934 | -0.0289 |
| **Upper water during NEM** |  |  |  |  |  |  |
| Depth | 0.1973 | 0.1973 | 0.3968 | 0.3968 | 0.0589 | 0.0589 |
| Temperature | 0.1060 | 0.1060 | 0.6054 | 0.6054 | 0.0236^*^ | 0.0236 |
| Salinity | 0.3992 | 0.3992 | 0.4096 | 0.4096 | 0.3967 | 0.3967 |
| pH | 0.1674 | 0.1674 | 0.8380 | 0.8380 | 0.0900 | 0.0900 |
| DO | 0.0729 | 0.0729 | 0.2901 | 0.2901 | 0.0239^*^ | 0.0239 |
| Phosphate | 0.0910 | 0.0910 | 0.6340 | 0.6340 | 0.0135^*^ | 0.0135 |
| DIN | 0.1569 | 0.1569 | 0.7297 | 0.7297 | 0.0703 | 0.0703 |
| TOC | 0.9553 | 0.9553 | 0.4439 | 0.4439 | 0.7263 | 0.7263 |

SWM, Southwest monsoon; FIM, fall inter-monsoon; NEM, Northeast monsoon.

* *p* < 0.05; ** *p* < 0.01.


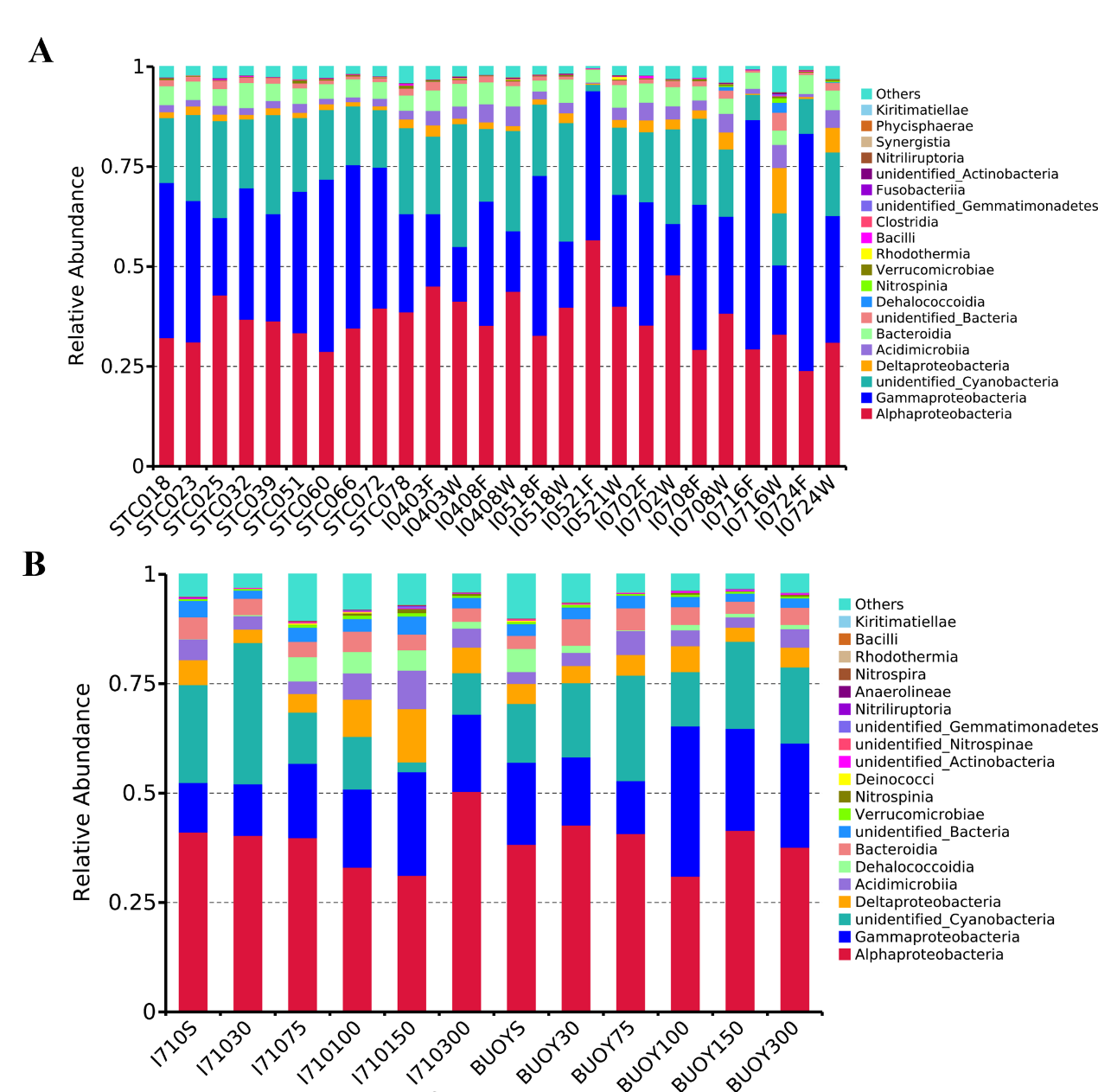


**FIGURE S1.** Relative abundance of bacterial sequences classified at the class level from (A) surface water of the Eastern Tropical Indian Ocean during the Southwest monsoon (SWM), the fall inter-monsoon (FIM) and the Northeast monsoon (NEM), respectively, and (B) in upper water (3 -300 m with six depths) during the Northeast monsoon (NEM).


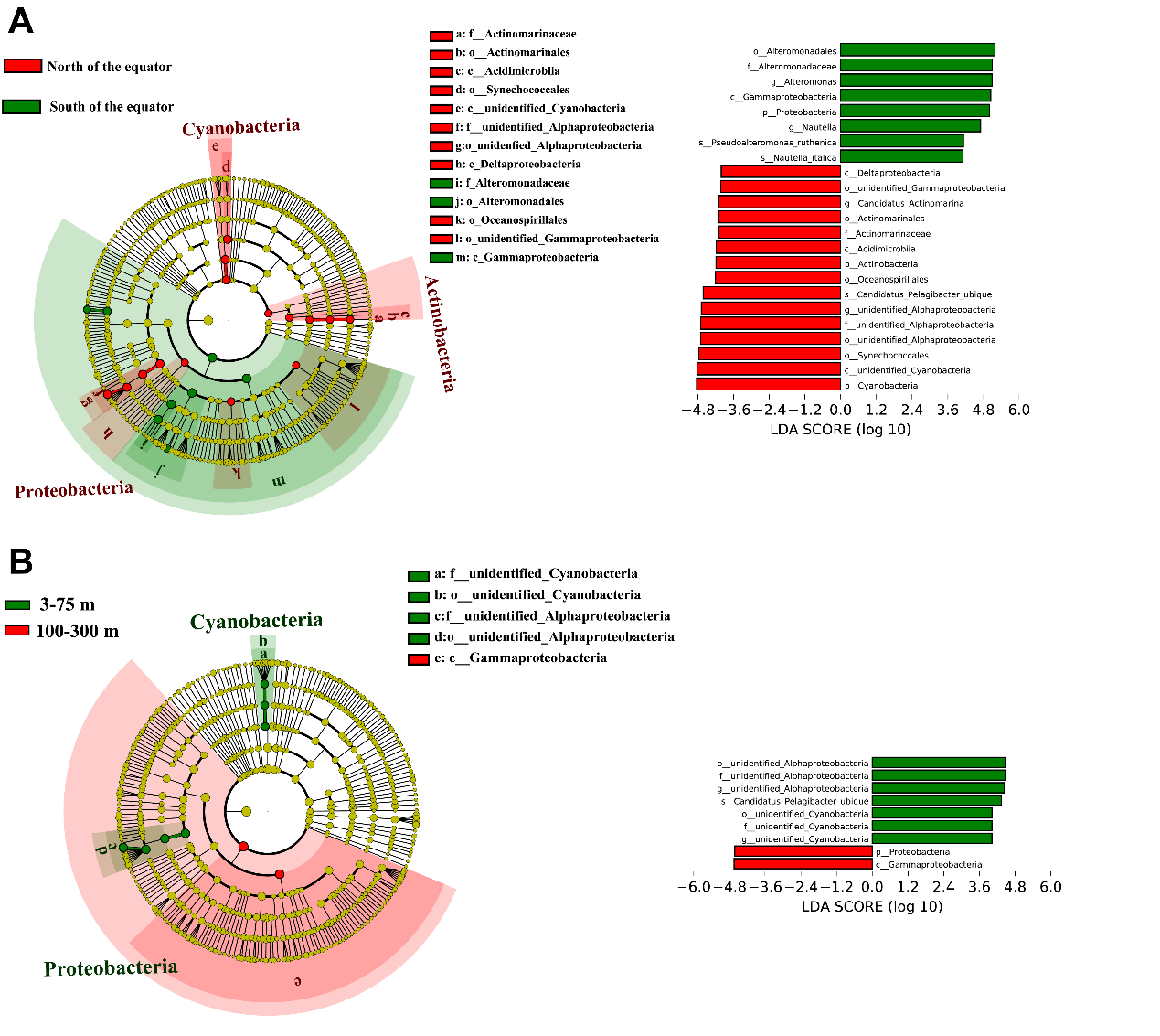


**FIGURE S2.** Cladograms showing the bacterial groups with significantly different abundance (A) in samples from north and south of the equator during the fall inter-monsoon (FIM), and (B) samples from 3 m to 75 m and from 100 m to 300 m according to LEfSe analysis, with a linear discriminant analysis (LDA) threshold of 4. The differentially abundant bacteria at different taxonomic levels among samples were highlighted by colored circles and shadings.


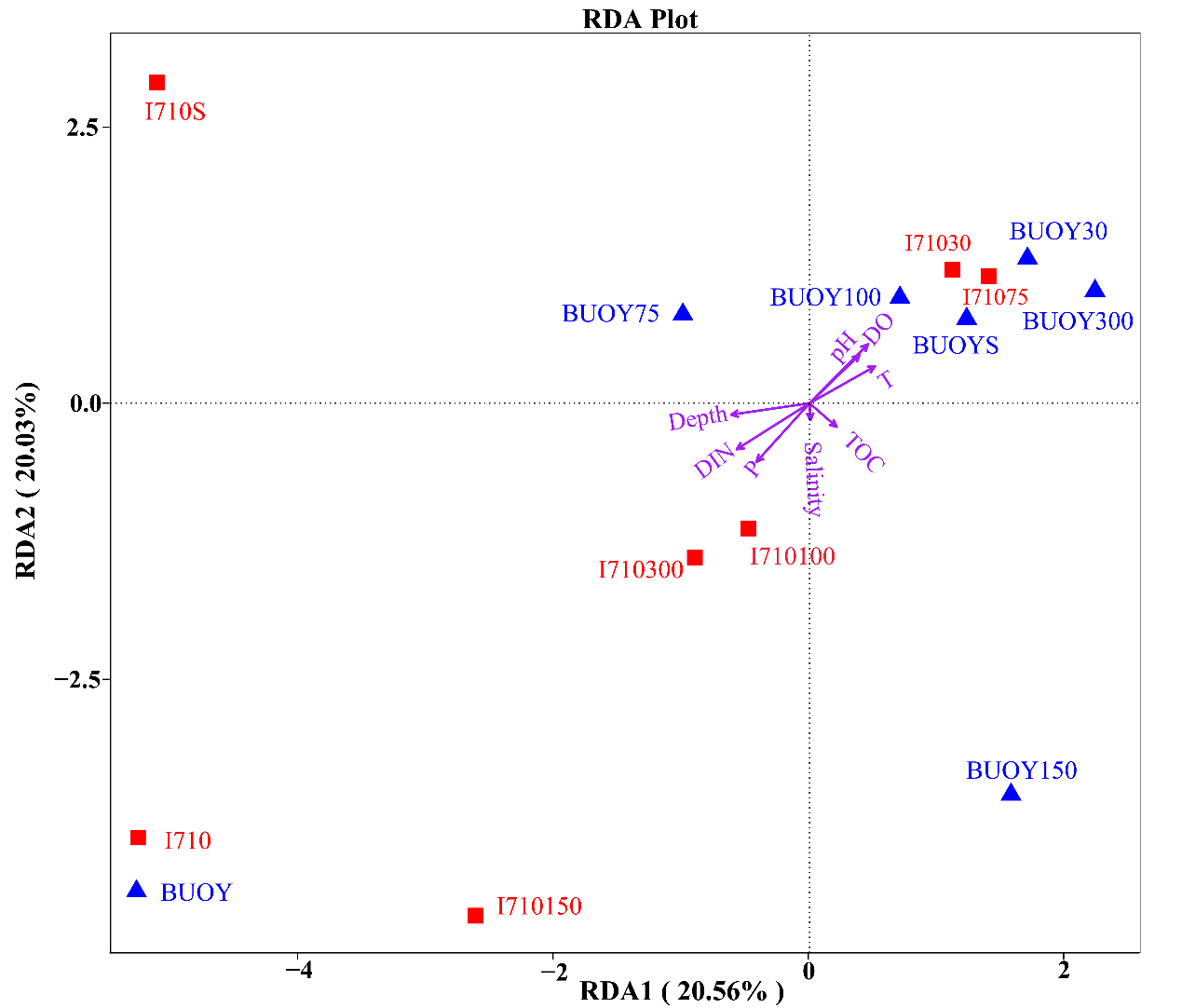


**FIGURE** **S3.** Redundancy discriminant analysis (RDA) for the relationship between bacterial communities and environmental factors in the upper water during the Northeast monsoon (NEM) in the Eastern Tropical Indian Ocean. Correlations between environmental variables and RDA axes were represented by the length and angle of arrows (environmental factors).


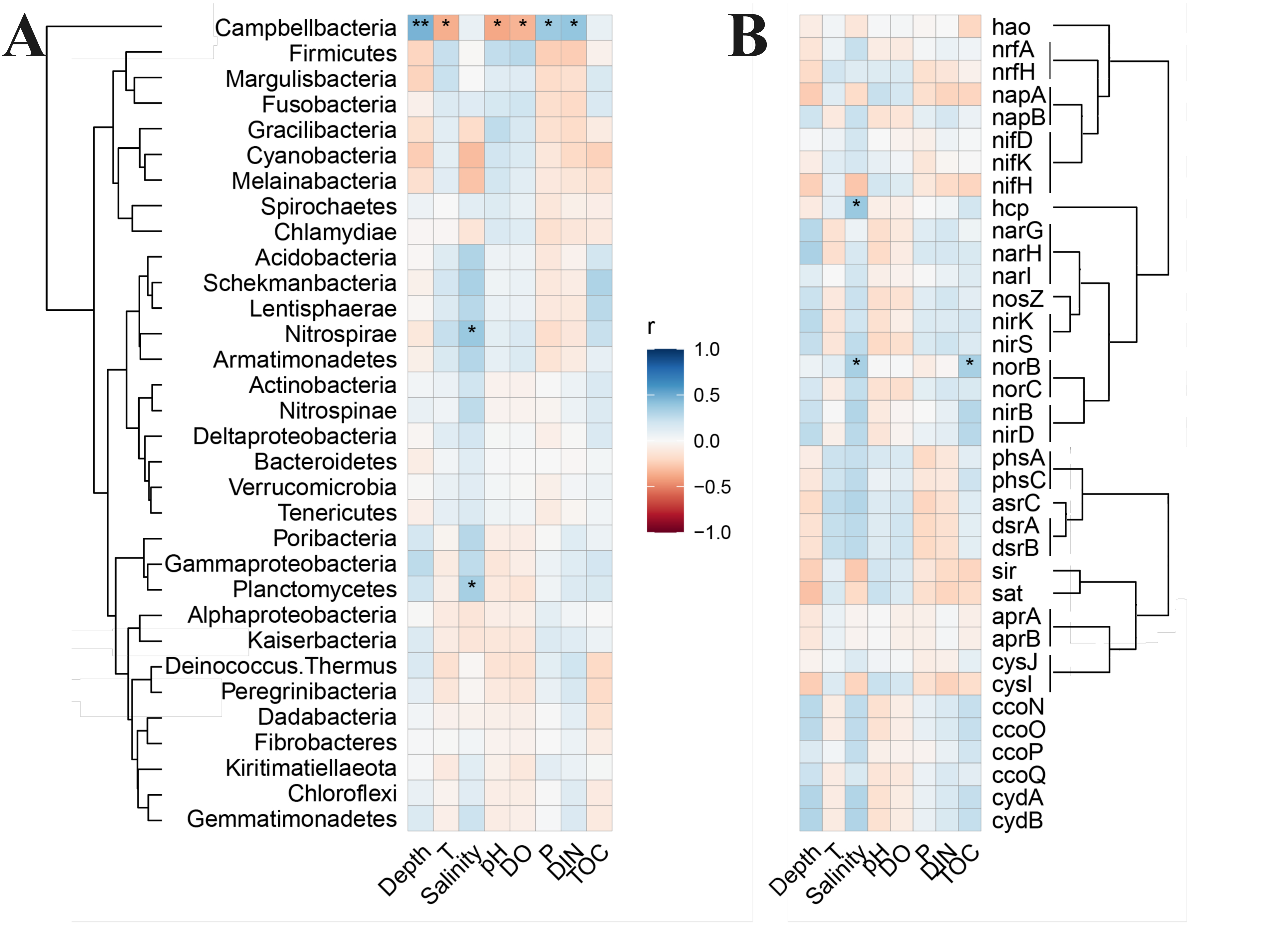


**FIGURE S4.** Pearson's correlation analysis between different bacterial taxa **(A)** or the predicted key functional marker genes related to nitrogen (N), sulfur (S), and oxygen (O) cycling **(B)** with environmental factors in upper water during the Northeast monsoon.

* *p* < 0.05, ** *p* < 0.01, *** *p* < 0.001.


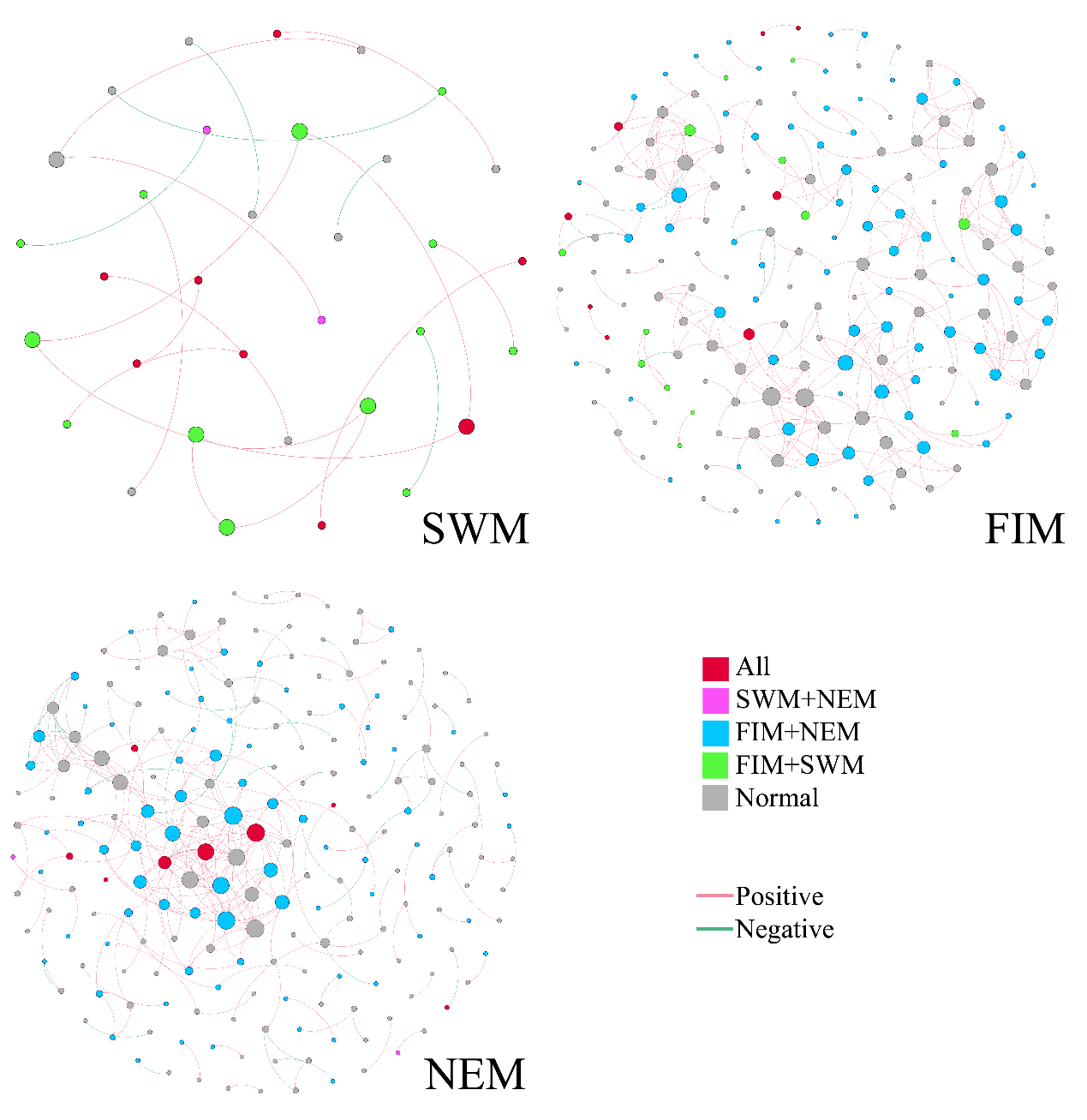


**FIGURE S5**. Co-occurrence networks of the bacterial communities during the Southwest monsoon (SWM), the fall inter-monsoon (FIM) and the Northeast monsoon (NEM), respectively, based on pairwise Spearman’s correlations between OTUs. The color in the nodes represents the seasons with the presence of the OTU. The size of each node is proportional to the number of connections. A red edge indicates a positive interaction between two individual nodes, while a green edge indicates a negative interaction. The position and taxonomy of each node are the same as Figure 8.


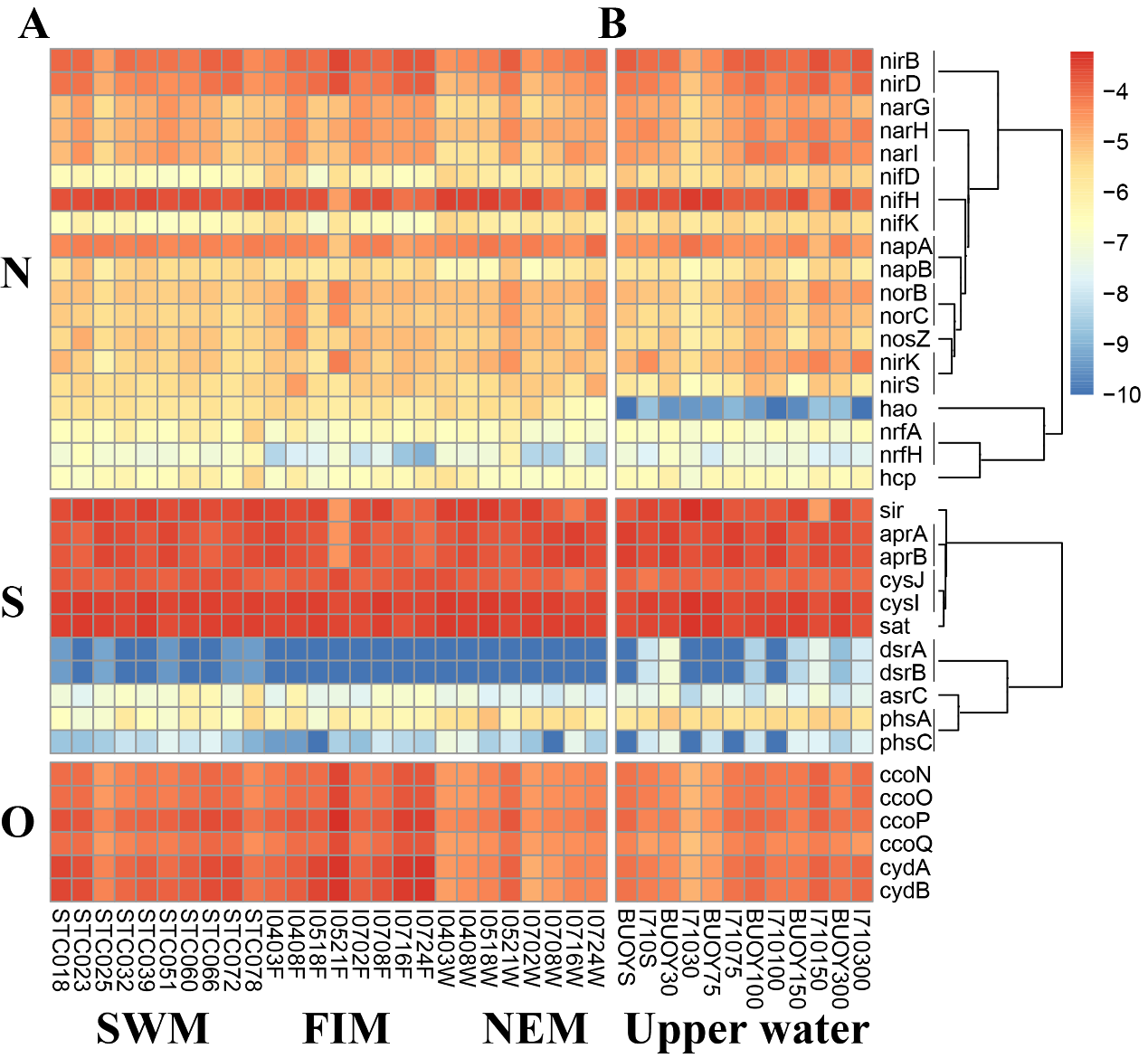


**FIGURE S6.** Relative abundance of the predicted key functional genes related to nitrogen (N), sulfur (S) and oxygen (O) cycling (A) across surface samples during the Southwest monsoon (SWM), the fall inter-monsoon (FIM) and the Northeast monsoon (NEM), respectively and (B) in upper water during the Northeast monsoon (NEM) in the Eastern Tropical Indian Ocean
